# Knowledge through social networks: Accuracy, error, and polarisation

**DOI:** 10.1371/journal.pone.0294815

**Published:** 2024-01-03

**Authors:** Ulrike Hahn, Christoph Merdes, Momme von Sydow

**Affiliations:** 1 Department of Psychological Sciences, Birkbeck, University of London, London, United Kingdom; 2 MCMP, Ludwig-Maximilians-Universitaet, Munich, Germany; 3 Interdisciplinary Centre for Ethics, Jagiellonian University Cracow, Cracow, Poland; AGH University of Krakow: Akademia Gorniczo-Hutnicza im Stanislawa Staszica w Krakowie, POLAND

## Abstract

This paper examines the fundamental problem of testimony. Much of what we believe to know we know in good part, or even entirely, through the testimony of others. The problem with testimony is that we often have very little on which to base estimates of the accuracy of our sources. Simulations with otherwise optimal agents examine the impact of this for the accuracy of our beliefs about the world. It is demonstrated both where social networks of information dissemination help and where they hinder. Most importantly, it is shown that both social networks and a common strategy for gauging the accuracy of our sources give rise to polarisation even for entirely accuracy motivated agents. Crucially these two factors interact, amplifying one another’s negative consequences, and this side effect of communication in a social network increases with network size. This suggests a new causal mechanism by which social media may have fostered the increase in polarisation currently observed in many parts of the world.

## Knowledge, testimony, and social networks

Much of what we believe to know is based, partly or wholly, on the testimony of others: Is the Earth round? Is anthropogenic climate change happening? Is Elvis Presley dead? Has crime gone up? Each of these questions, large or small, involves a claim for which one likely possesses *some* relevant data that is based on personal observation of the world such as the curvature of the horizon at sea, local weather in the past years, experience of a relationship between age and death, signs of vehicle break-in on the street and so on. But to a considerable extent, our beliefs about these issues are formed by *reports* we receive from others. These reports may involve further evidence or may simply be assertions that the claim itself is true. In either case, we rely critically on others and their reliability. Only rather recently has the social basis of much of our knowledge been recognized. The social influence on our beliefs raises important questions not just about how we actually respond to information received from others, but also about how we should respond. If we receive information from someone we know little about, it seems reasonable to assume that this person is not always 100% accurate in what they claim. In fact, even the most diligent, expert, and trustworthy, among us get things wrong and tell others things that turn out not to be true. How should we factor in this less than perfect reliability/accuracy? And what are the consequences of the fact that our sources are less than perfectly reliable? And is any of this impacted by the fact that our sources likely communicate not just with us, but are part of wider networks of communication? These are the questions addressed in this paper. Specifically, agent-based simulations demonstrate the impact of the reliability of our sources, the fundamental strategies we use to estimate that reliability, and the social networks that the communicating agents are part of. Implications for understanding the impact of changes to our information environment, in particular the advent of social media, are discussed.

## Coming to know the accuracy of our sources

To appreciate the problem of how we should deal with the less than perfect accuracy of testimony, it helps to start with a simple case of non-testimonial evidence and its incorporation into our beliefs, stripping away, for a moment, any social context. Imagine, for example, a new pregnancy test. Before you bring this new test to market you try it on lots of pregnant (and non-pregnant!) women. Then, based on comparison between test result and eventual outcome, you record the numbers of ‘true positives’ (cases where the test indicated a pregnancy and the woman did, in fact, turn out to be pregnant) and ‘false positives’ (cases where the test indicated a pregnancy, but no pregnancy was found). These numbers provide a best estimate of the test characteristics; that best estimate can then be used to optimally calculate a degree of belief in pregnancy given the results of the test, *P(h|e)*, using Bayes’ rule:

$$P(h|e)=\frac{P(h)P(e|h)}{P(h)P(e|h)+P(\neg h)P(e|\neg h)}$$ Eq. 1

Specifically, you would use the recorded ‘true positive’ or ‘hit’ rate, as your best estimate of a key quantity in Bayes’ rule: the so-called likelihood *P*(*e*|*h*). It represents the probability of observing the evidence (here, a positive result on the pregnancy test), given that the hypothesis is true (here, that the woman is pregnant). Likewise, you would use the recorded false positive rate as your best estimate of *P*(*e*|¬*h*)–the probability of a false positive given the hypothesis is false (in this case, the test erroneously suggesting pregnancy). The ratio of *P*(*e*|*h*), or hit rate, divided by *P*(*e*|¬*h*), or false positive rate, is known as the likelihood ratio. It is a natural measure of the quality (‘diagnosticity’) of the evidence–that is, its informativeness regarding the hypothesis or claim in question: the larger the hit rate relative to the false positive rate, the more accurate the test.

Where these evidence characteristics are known, revising one’s beliefs in light of evidence using Bayes’ rule (in other words, “being Bayesian”) is demonstrably optimal in the sense that it will minimise the expected inaccuracy of one’s beliefs [1,2], assuming that (in)accuracy is measured by a common measure of (in)accuracy (effectively the mean squared error, known in that literature as the so-called Brier score; [3]).

Such a frequency-based strategy that monitors the co-occurrence of evidence and eventual outcomes has been called an *outcome-based strategy* for estimating evidence quality [4]. It underlies not just the actual certification of medical tests, but many real-world estimates of evidence quality such as forensic tests [5] or forecasting models. Outcome-based estimates will be more accurate the more evidence/outcome pairings one can observe, and less accurate estimates of evidence quality will lead to less accurate beliefs (see [4] for details and visualization). However, the accuracy costs may be small in practice, particularly in situations where one has access to a lot of evidence: even if one noticeably mis-estimates the quality of one’s evidence, beliefs will converge to the truth as long as one is right about the *qualitative impact* of the evidence (that is, whether it counts as evidence for or against) and individual pieces of evidence are independent (see [4] and references therein).

However, outcome-based strategies will work only where there is an outcome (e.g., pregnancy) that occurs repeatedly *and* has a correlation with potential evidence (e.g., pregnancy test result) that can be observed. Many real-world claims of interest do not qualify here because they concern singular events (“did Oswald murder Kennedy?”). At the same time, many cases of testimonial evidence do not involve informants whose accuracy with respect to the issue at hand we are able to estimate on past performance. In fact, in many cases, both these difficulties come together: In a legal trial, for example, we are only concerned with the one case before the court and we will likely only ever hear the witness speaking to this one case. How can one estimate the diagnosticity of that witness testimony?

In such circumstances, one may try to estimate the witness’s reliability by drawing on *indirect* evidence that is ultimately outcome-based: for example, there may be speech patterns, or patterns of eye gaze, that have been shown (through observations of evidence/outcome pairings) to provide cues as to whether people, in general, are lying (e.g., [6]). Such an inference is still grounded in observations of past outcomes, albeit indirectly.

However, even that might not be possible in contexts where testimonial evidence comes from sources with whom we have no direct, personal interaction (e.g., ‘climate scientists’). In this case, we have two possible strategies left. The first is to simply *assume* a particular degree of reliability (diagnosticity) for such unknown sources: in particular, one might simply assume that the source is moderately likely to be right in confirming or rejecting a hypothesis (say, with probability *p* = .66, assuming symmetry, here and in the following, whereby people are as good at providing evidence for the hypothesis when it is true, as they are at providing evidence against the hypothesis when it is false). Such an assumption has a general basis in reality, in that we would not bother with human communication if, on average, people weren’t at least somewhat more likely to be right than wrong. The second strategy is to try to estimate the reliability of a source on the basis of how expected the content of their evidence is: that is, one uses one’s present (uncertain) degree of belief in the claim in question to adjust one’s belief about the reliability of the evidence reporting source. In other words, one tries to assess the reliability of, say, the witness in the trial mentioned above, on the basis of how plausible the statement is that she is making.

The simple logic of this kind of strategy runs as follows: if you say to me something that I think is unlikely to be true, I will nevertheless increase my belief in what it is you are claiming, but I will also decrease my belief in your reliability. If you tell me that the Earth is flat, this strategy will make me think that this is a tiny bit more likely to be true, but it will also make me think that you are less reliable than I had previously thought. This strategy has been labelled ‘expectation’ or ‘belief-based’ updating (see [4,7]) because it is the mismatch between the evidence expected, given what one presently believes is likely to be true (but does not actually *know* to be true!), and the evidence received that drives the reliability estimate. This strategy seems so intuitive that philosophers have considered it to be a rational, normative solution to the problem of testimonial evidence [8–10]. At the same time, there is experimental evidence that people actually do make use of such a strategy [7,11].

Hahn and colleagues [4] compared the performance of a fixed-trust and an expectation-based update strategy through simulations that involved information received from a single source. In the present paper, we use these two strategies to examine the influence of social networks on two fundamental aspects of beliefs: accuracy and belief polarisation within a community.

### Social networks, (in-)accuracy, and polarisation

In our simulations, we examine the behavior of a simple Bayesian agent (first proposed by Olsson, [8]) who formally implements the strategy intuitively outlined with the ‘flat Earth’ example (see Supp. Mat. A. for the full, formal definition of this strategy). In other words, this agent treats the match or mismatch between a piece of evidence and his present beliefs about the truth or falsity of the underlying hypothesis as evidence with which to update beliefs about the reliability of the source. This agent is compared to a fixed trust agent, who simply assumes that sources are moderately accurate (in our simulations *p* = .66) and does not seek to modify this belief.

Agents in the simulation may receive evidence from both ‘the world’ (reflecting the fact that we may obtain real world evidence through our own observation or experimentation) and from other agents. At stake in their world is a single claim, and the sources from which they receive evidence assert the truth or falsity of that claim, or, on a particular trial remain silent. On receipt of a piece of evidence, agents revise (via Bayes’ rule) their beliefs in light of that evidence and this revised belief forms the basis of their communication with others at the next time step (by computational necessity, agents assume their sources are independent; in other words, they are a type of naïve Bayesian agent; see, e.g., [12]). In this way, the simulated society dynamically modifies its beliefs over time. Crucially, there is a ‘ground truth’ on any given run of the model, such that the underlying claim is either true or false; hence the accuracy of the simulated agents can be measured with respect to that ground truth. Key factors we will vary are the true quality of the evidence, the perceived quality of the evidence, and whether or not the agents communicate with others in a social network.

The model is a reimplementation (in NetLogo, see Supp Mat. A) of Olsson’s [8,9] social network model. The type of network and the network size can be varied. We focus here on small-world networks [13]. Small world networks are a type of network structure found in many social and biological networks; specifically, small world networks are characterized by comparatively short paths between nodes in the network (‘five degrees of separation’) and comparatively high clustering even though link density is fairly low. The topology of Facebook, for example, has been shown to exhibit these key properties [14]. To study the effect of network size, we increased the number of nodes in the network, while keeping constant the number of direct neighbours for each node.

The running of the model is stochastic. At each time step in the model, agents receive evidence from the world and/or other agents to whom they are linked; the underlying probability of receiving evidence from either is a free parameter in the model. When communicating, agents assert the claim in question as true (or false) if their present belief lies beyond a “threshold of assertion”; otherwise they remain silent. Agents start agnostic with respect to the truth or falsity of the claim at issue (i.e., their initial belief is *p* = .5). The model is then run for 50 time steps, at which point agents’ beliefs are assessed. This run length has worked well in past research [4,15] and allows direct comparison with that work. For readers interested in the micro-dynamics of how agent beliefs evolve, sample runs are included in Supp. Mat. SB, and [16] provides detailed analyses.

Finally, in order to isolate the effects of communication, each agent in our simulation is ‘shadowed’ by an agent (referred to as ‘shadow agents’ in the following), initialised with the same prior and trust values, that subsequently receives exactly the same evidence from the world as the shadowed agent, but does not participate in communication.

Importantly, our simulations factorially combine the quality of evidence coming from the world, network size, whether agents make use of expectation-based updating or simply adopt a fixed level of trust, and whether or not they participate in communication. As a result of these manipulations, the simulations provide insight into causal effects.

**Fig 1:**
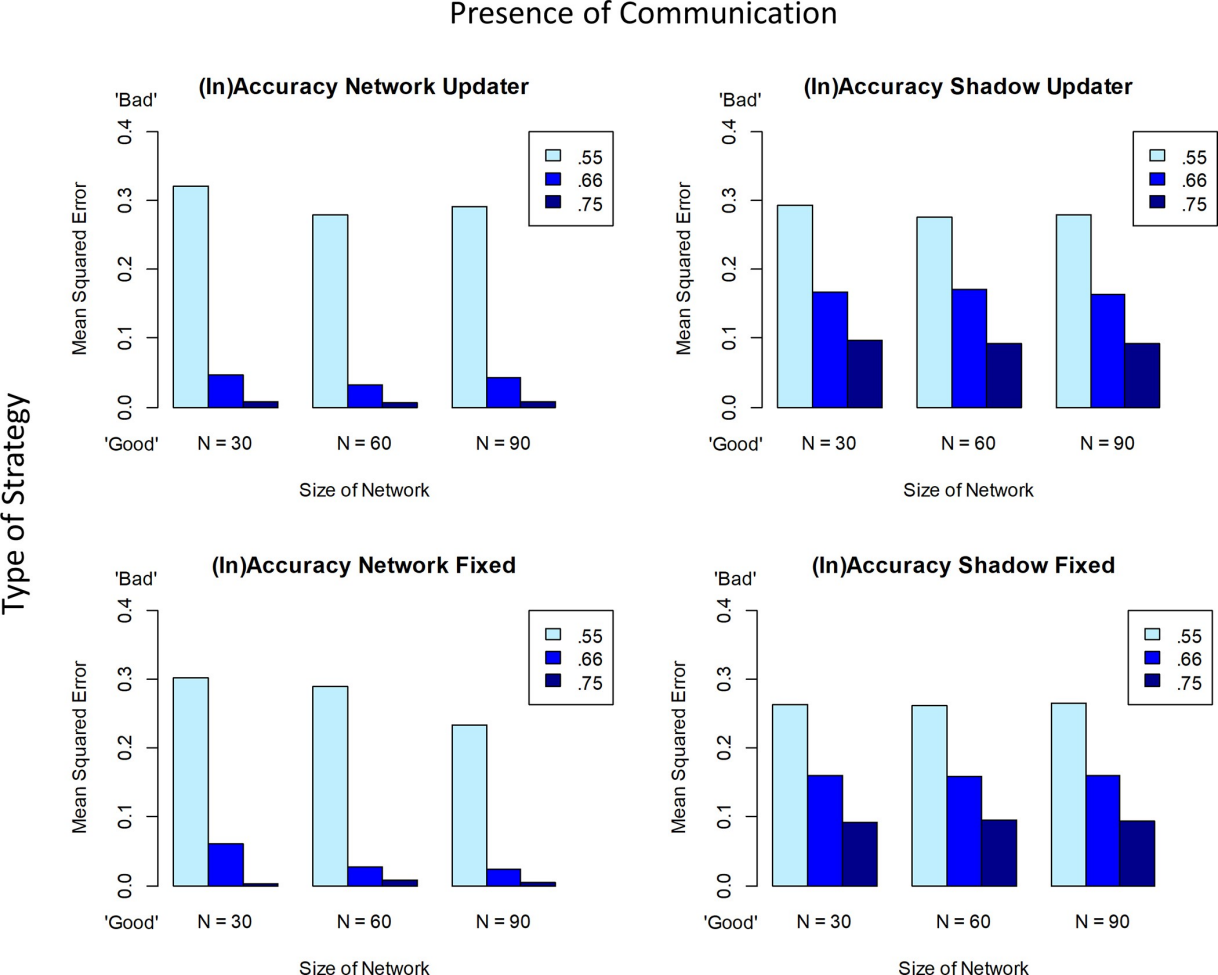
Mean accuracy values (mean squared errors) for the updating vs. the fixed-trust agents in a network of either communicating agents or corresponding non-communicating shadow agents. For each of these four cases, the respective graphs show the results for a different number of agents (*N* = 30, 60, 90) and for varied degrees of reliability of the data reports (*p* = .55, .66, .75). As parameters we used a belief prior of *p*_*sub*_(*h*) = .5 and, for the updaters a positive trust prior probability distribution with *E*(trust) = .66 (from a Beta distribution of *beta*(2,1)) and for the fixed-trust agent a fixed value of .66. The probability that the hypothesis was actually true on any given simulation run was *p*_*obj*_(*h*) = .6. Global activity (probability for each agent of receiving information from the world) was *p* = .1, the threshold of assentation was exceeded for *p*_*sub*_(*h*) > .8, with a *p* = .25 chance of communication if an agent’s belief passed this threshold. For each data point we ran the model for 50 time steps and averaged over 100 model runs. We employed a small world network with *k* = 2 and a rewiring probability of .2. More detailed information on the meaning of these parameters is found in Appendix B.

Fig 1 below shows the basic simulation results. With respect to accuracy, the figure shows several broad patterns. First, higher quality evidence (unsurprisingly) leads to more accurate beliefs. The bars of different shades represent different levels of accuracy of the evidence coming from the world, with darker bars representing objectively more reliable evidence (light, medium and dark blue for accuracies of *p* = .55, *p* = .66, and *p* = .75 respectively). Specifically, a level of accuracy/reliability of *p* = .55 represents the fact that ‘the world’ is dispensing evidence that the claim in question is true or false with accuracy *p*(e|h) = *p*(¬*e*|¬*h*) = .55 (see also [4] and Supp. Mat. D1 on why this symmetry assumption does not seem to impact the generality of the results). As seen in Fig 1, the darker the bars the lower the error, across all conditions. Second, there is surprisingly little difference between the expectation-based update strategy and fixed-trust agent with respect to accuracy (top row vs. bottom row). It may seem counterintuitive that an agent who doesn’t even try to gauge accuracy performs this well, but the results here mirror those of the extensive analyses of individual agents reported in [4]. Third, being in a network and communicating with others is not always beneficial in these simulations (left column networked agents vs. right column ‘shadow agents’). In general, the extent to which communication helps (or even hinders) depends on the extent to which communication conveys additional, true, information or merely serves to homogenise opinion. This will depend both on the individual accuracies involved and on the network structure, as has been shown both in human behavioral experiments [17,18] and simulations [19]. Finally, we note that accuracy does not seem to be (systematically) affected by network size.

**Fig 2:**
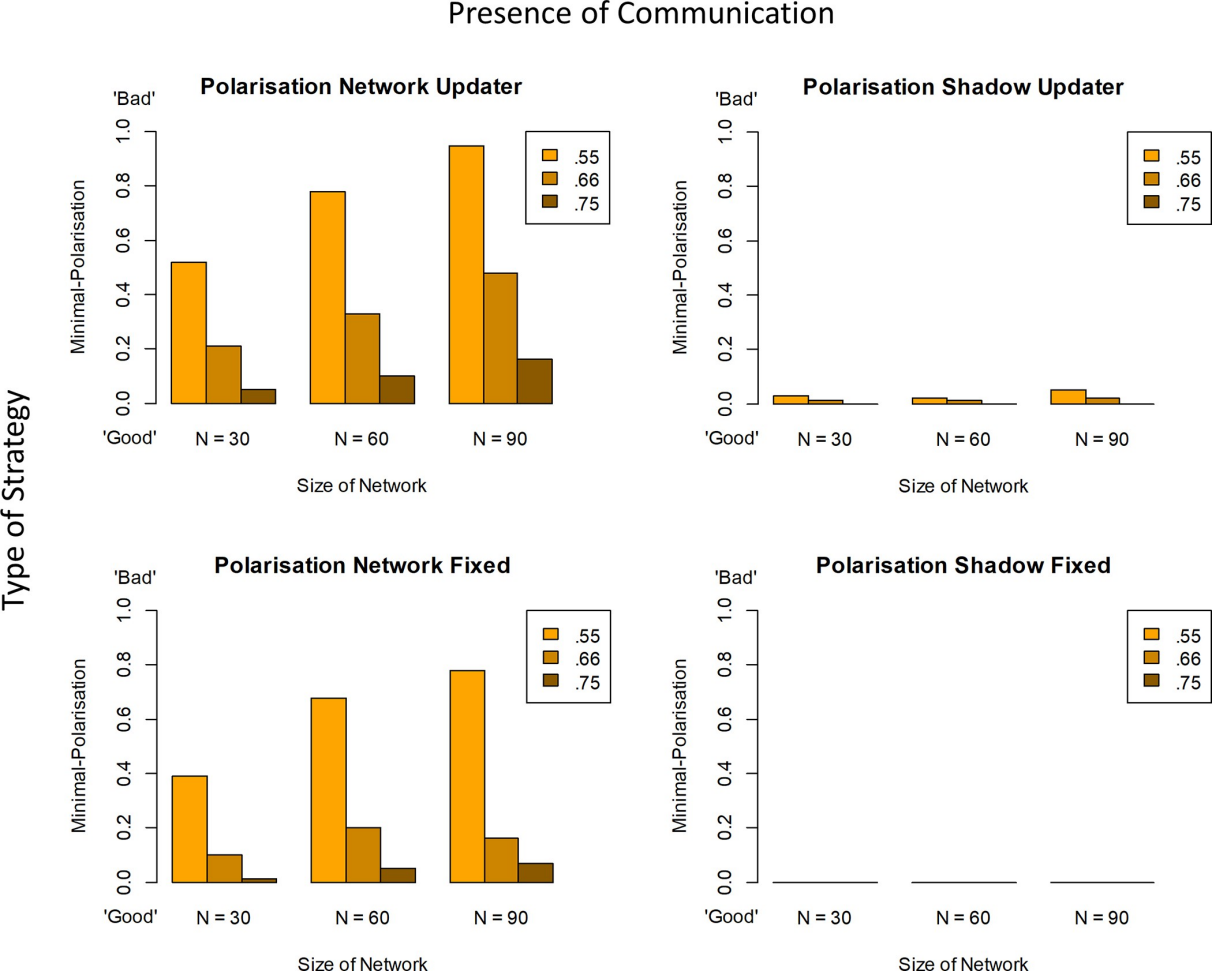
Minimal polarisation, formalizing the existence of at least one agent believing strongly the hypothesis *h* (with *P*_*sub*_(*h*) > .999) and at least one agent believing strongly non-*h* (with *P*_*sub*_(*h*) < .001) within a single population at the end of the run of the model. (See Fig 1 for parameter details and corresponding accuracy results).

Fig 2 shows the corresponding results for group polarisation. Displayed are the proportions of simulated populations which end up “polarised” in the sense that they simultaneously contain *both* agents that are maximally convinced the claim is true and agents who are maximally convinced that it is false. Once again, the quality of evidence coming into the network from the world matters: the less accurate that information (lighter bars) the more polarisation ensues. Second, expectation-based update *in and of itself* fosters polarisation, as can be seen from the comparison between the two types of shadow agents (which show *no* polarisation for fixed-trust agents). Independently of this, communication promotes polarisation (for both expectation-based update and fixed-trust agent, polarisation is higher for networked than for shadow agents). And, crucially, the two independent sources of polarisation, expectation-based update and network communication, may interact negatively, giving rise to super-additive effects. Disturbingly, this interaction is exacerbated by increases in network size as Fig 2 shows. Here, the fact that *no* size-based increase is observed in the fixed-trust shadow agents indicates clearly that polarisation doesn’t increase merely because the society itself gets larger (as seen in [20]), but rather increases because of communication.

**Fig 3:**
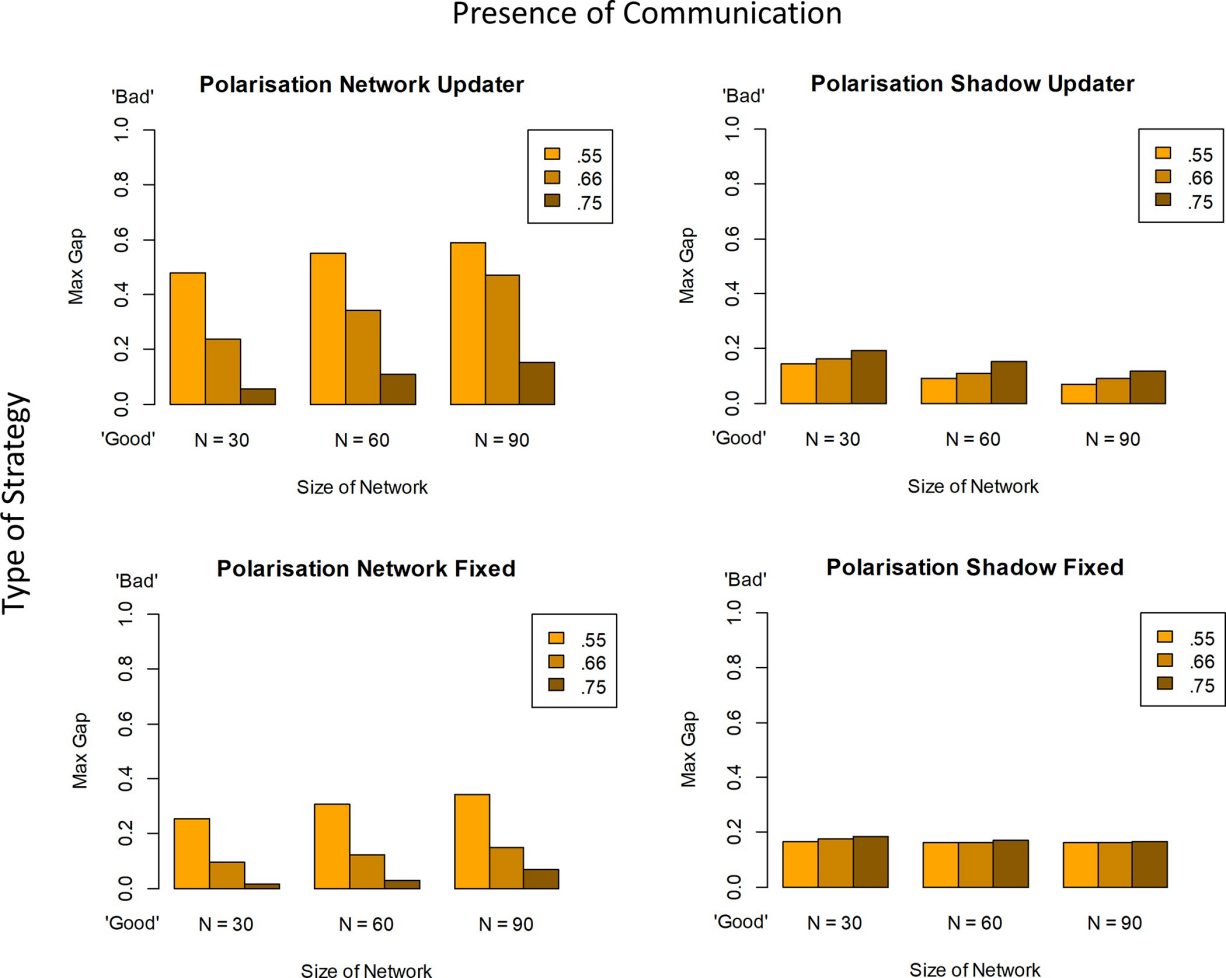
Maximal-gap polarisation [Exp. 5] measures the span of the largest ‘gap’ in degrees of belief separating groups of agents.

The measure of polarisation underlying Fig 2 measures only whether there are extreme agents at both ends of the belief spectrum within a given population. This is a “minimal” measure of polarisation in a number of ways. It is imperfect in that a uniform distribution of beliefs would also count as ‘polarised’ on this measure, because of the presence of ‘extremists’; yet such a population encompasses agents with similar, adjacent beliefs right across the entire spectrum. In other words, the measure does not guarantee the existence of distinct, separated groups that face each other across a chasm. However, in the specific context of these simulations, one gets agents with extreme beliefs only because beliefs have separated out given that all agents start with same agnostic prior of .5 –and when they do, they generally leave ‘gaps’ (for sample plots of final belief distributions see S1–S4 Figs). This degree of separation can be measured directly by establishing the largest (unpopulated) “gap” in degrees of belief between agents. Fig 3 shows this alternative measure of polarisation and confirms all of the key results.

Finally, S5 Fig, shows a corresponding figure with a further, third, measure (the standard deviation as in [4,21]), and draws out additional inferences about the data this affords in a theoretical note that explains and justifies further our approach to measuring polarisation (for a range of other possible measures of polarisation see also, [22–24] as well as references therein). To conclude, in the present context, assessing the mere presence of opposing ‘extremists’ provides a simple and robust measure of polarisation.

By polarisation, as discussed so far, we just mean ‘separateness’–that is, the existence of a particular distribution of beliefs, not a process. In the psychology literature, polarisation is also used to refer to a *process*, whereby people exposed to the same evidence, move in opposite directions [25]. Our “update agents” may also exhibit this behavior, because two such agents can have opposing views on the reliability of a single source, suggesting that seeming “biased assimilation” may occur for entirely ‘rational’ reasons (see also [26]). Fixed-trust agents, by contrast, will never show opposing responses to the same evidence, because they have the same degree of trust by design. The differences in the degree to which polarised distributions arise from these two types of agents thus also reflect this underlying difference in process.

The contributions of both communication and the update strategy as a means for determining trust, coupled with the exacerbating effect of network size have obvious policy implications. However, before considering these, readers will wish to know how robust these effects are. That these broad effects hold across changes to other parameters of the model is shown in the supplementary graphs for varying threshold of assertions S7, S8 and S9 Figs.

**Fig 4:**
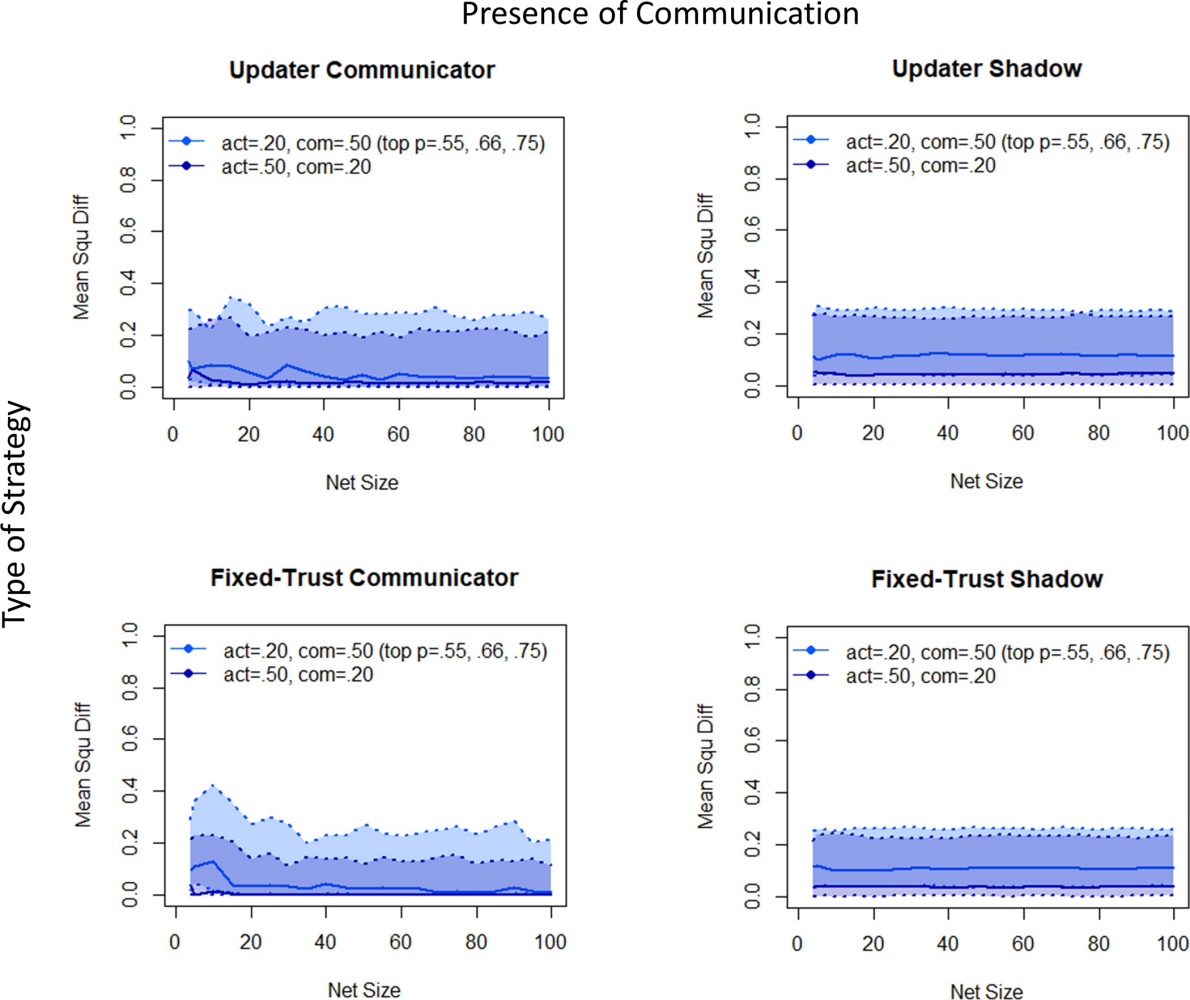
Accuracy values varied over network size. We started with n = 4 as the smallest network that can contain two ‘groups’ (n > 1); this is followed by n = 5 to n = 100, modelling all multiples of 5. Displayed are results for either a high relatively high communication condition with global activity of .20 and a communication probability of .50 or a relatively low communication condition with global activity of .50 and a communication probability of .20. For both conditions, we additionally varied the objective reliability of data from the world, with *p* = .55, .66, .75. The error scores for each of these obj. likelihoods is represented by line type: The upper dotted line with greater deviation from the truth corresponds to *p* = .55, the continuous line in the middle represents *p* = .66, and the lowest dotted *p* = .75. The area between the two dotted lines is shaded in the color of the corresponding condition (high communication/low global activity vs. low communication/high global activity). Overlapping colors reflect regions of overlap.

**Fig 5:**
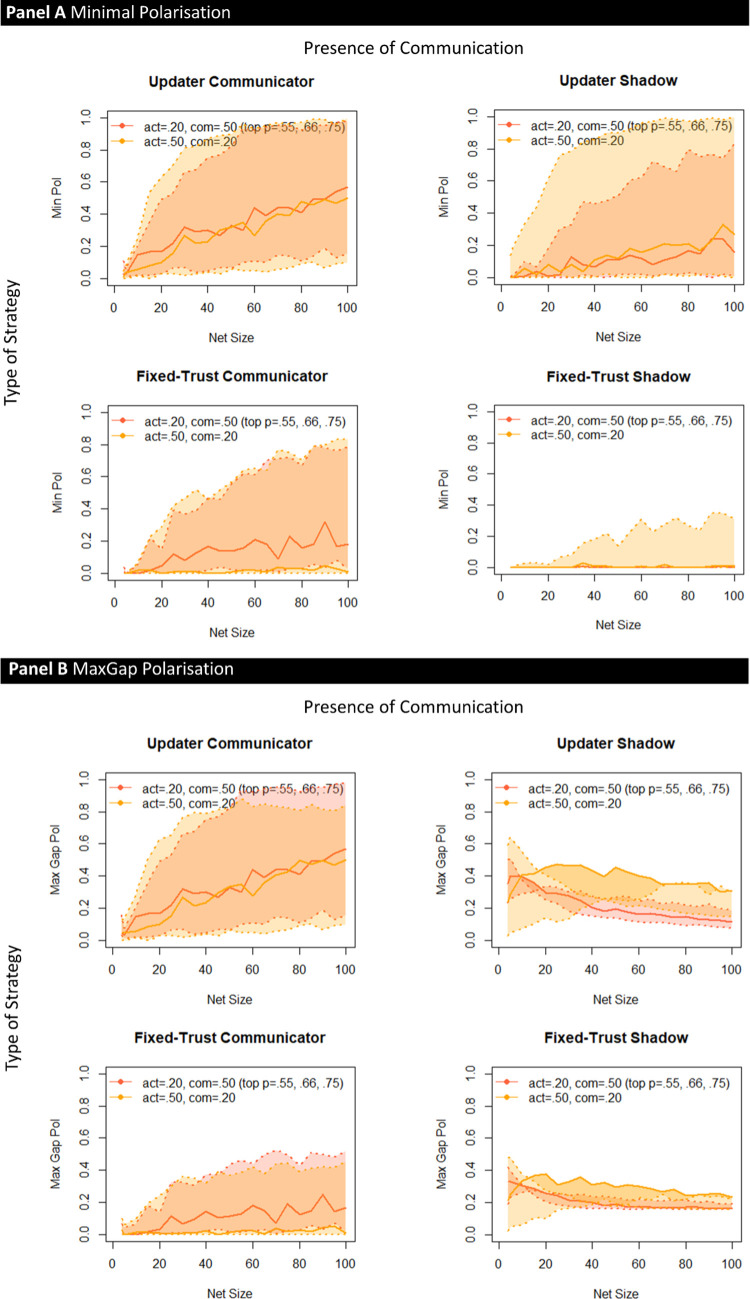
Corresponding polarisation plots. The top four panels show the “minimal” polarisation measure (mere existence of “extremists” on both sides within a single population), the bottom four show the size of the largest “gap” in degrees of belief between the two sub-populations. See text for further explanation. The upper dotted line with greater polarisation corresponds to *p* = .55, the continuous line in the middle represents *p* = .66, the lowest dotted to *p* = .75. The area between the two dotted lines is shaded in the corresponding color.

More interesting is the relationship between communication and information from the world. Given that higher quality evidence increases accuracy while reducing polarisation (Figs 1 and 2, above), one would expect that altering the relationship between information from the world and communication would have some impact on results. Fig 4 (accuracy) and Fig 5 (polarisation) below show the results of shifting that relationship. Light blue shading represents the parameters similar to those underlying Figs 1–3, with relatively high communication and low amounts of data from the world (determined in the model by the level of “activity” from the world); the dark blue shading represents increased information from the world and decreased communication. As expected, accuracy improves with more information from the world (in keeping with the lower levels of error for higher quality evidence), see Fig 4. Under these circumstances, communication is beneficial across the range of network sizes; only for low activity of the world and high communication combined with a low reliability (*p* = .55) is this advantage not systematic.

The impact on polarisation of increasing evidence from the world while decreasing communication (Fig 5), however, is complex, with multiple interactions between evidence quality, presence or absence of communication, and trust strategy. The fundamental pattern of greater polarisation given communication, and greater polarisation for the update agent is retained, however, as is the increase in polarisation as a function of the size of the communication network.

In short, the finding that both expectation-based updating and communication give rise to polarisation, and that their negative effect increases with network size seems robust over changes to key parameters of the model.

One final, potentially important, factor of interest with respect to real-world networks is the extent to which these effects may be moderated by network structure. As noted above, network structure has been found to affect accuracy in real-world social networks (see e.g., [17,18]) and network structure has been shown to have effects on accuracy in the modelling framework used here even for networks of the same link density [19]; so, to probe this further we manipulated the rewiring probability, *p*_*r*_, that determines network structure. This parameter allows one to modify the network topology from regular lattice (at *p*_*r*_ = 0), through small world network, to random graph (at *p*_*r*_ = 1). Random networks have been the ‘fruit fly’ of network science [27] and differ from small world networks in relevant topological characteristics such as the degree of clustering and the average path length.

**Fig 6:**
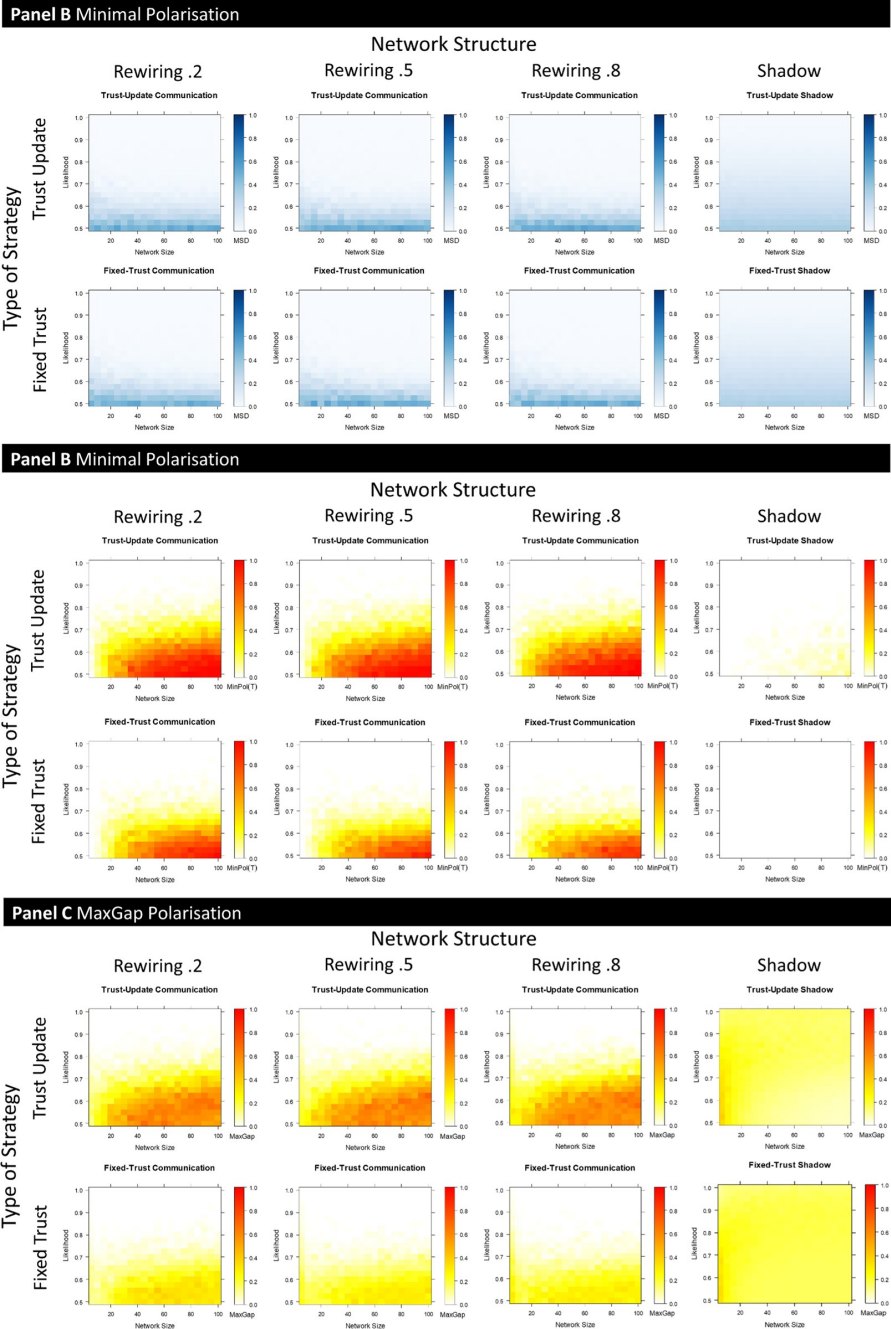
Accuracy and polarisation plots across the combinations of network size (*x*-axis) and evidence quality (*y*-axis). Other parameters correspond to those given for Fig 1.

Fig 6 shows the results of these simulations for both (in)accuracy (mean squared error) and polarisation. Each chart not only varies the network size but now does so over the range of possible objective likelihoods (*y*-axis). Each data point in the heatmap thus represents a combination of likelihood and net size (for the given rewiring parameter) and is based on 100 model runs (once again over 50 steps), meaning each chart summarises 44,100 runs). The third dimension of the heatmaps, color, represents the dependent variables (in)accuracy (mean squared error), minimal polarisation and maximal polarisation, respectively.

As can be seen, accuracy is not meaningfully affected by structure (though we have found differences between random and small world networks in our work elsewhere): there is, at best, a hint of an increase in accuracy with network size, but whatever differences there are, are dominated by being in a network versus not being in a network, as seen by the comparison between networked and shadow agents. Those comparisons show a clear interaction to the effect that being in a network is beneficial for high objective likelihoods but detrimental for lower ones.

The contrast between network and no network also dominates the degree of polarisation observed. Looking across different structures, the broad range of sizes and the range of objective likelihoods the key observations thus far are confirmed: expectation-based updating causes polarisation, but communication across a social network is far more influential. The ability to distrust one’s neighbors is not a necessary ingredient for polarisation to arise. There is also a clear effect of network size, not seen for accuracy, that allows extrapolation to larger networks. Finally, there are also indications that, for polarisation, structure matters more. While there is no real difference for the update agent across the different values of the rewiring parameter, there does seem to be a trend for the fixed-trust agent, whereby polarisation decreases as re-wiring probability increases. The change in network structure that accompanies this change in re-wire probability is a decrease in clustering (with the bulk of the drop happening between 0 and .5). The fact that polarisation decreases somewhat for the fixed-trust agent, but not for the update agent, in this range highlights the way clustering plays a greater role in maintaining “minority” views for the fixed-trust agent, than it does for the update agent, given that only the latter can come to distrust others. Fixed-trust agents, by contrast, will need to rely on a supportive neighbourhood of like-minded agents providing testimonial support in order to counter conflicting evidence from the world.

### The findings in context

Understanding group polarisation has been viewed as central to understanding a uniquely wide range of phenomena. In his monograph on polarisation, Sunstein [28] asserts that understanding polarisation.

‘offers large lessons about the behavior of consumers, interest groups, the real estate market, religious organizations, political parties, liberation movements, executive agencies, legislatures, racists, judicial panels, those who make peace, those who make war, and even nations as a whole’ [28, p. 3].

This is not just a bold claim. There is evidence for the impact of group polarisation on everything from risky decisions [29], through judicial panels [30], to the rise of ethnic tensions and war (e.g., [31,32]).

Given this potential breadth of application, it should come as no surprise that the term ‘polarisation’ may mean subtly different things in different contexts. Above, we have already distinguished polarisation as a property of a population and polarisation as a process. Bramson et al. [33] distinguish nine different, inter-related meanings; the reviews of [34,35] each distinguish three different forms.

Confusingly, the term polarisation is used both to indicate the divergence of opinions/beliefs within groups, as we have used the term here, and to indicate the move toward extremity of a single group opinion (see also [34]). These two distinct uses will often be at odds: the latter requires that the individuals within a group have come to hold extreme opinions (typically more extreme than the views they started with) and broadly agree on those opinions. The former requires that individuals have come to hold extreme opinions *and* are now at odds with one another on those views. Both uses of the term polarisation have been the focus of longstanding scientific interest, and both are subject to renewed societal concern. However, it is polarisation in the sense of (extreme) belief divergence that seems the core phenomenon of interest. When and why groups move consensually toward more extreme opinions is arguably of interest in large part because such groups are typically subgroups within larger populations that contain other subgroups from whom they now diverge–simply because consensus opinions are unlikely to ever be viewed as extreme.

In keeping with this, it is polarisation in the sense of belief divergence that has seen a recent resurgence in interest in response to perceived societal trends of increasing opinion divergence, tribalism and partisanship in Western liberal democracies (e.g., [28,36,37], and references therein; but see also e.g., [38,39], with evidence suggesting that party ties are now stronger than adherence to the social groups parties represent [40], and, in the U.S. elicit stronger responses than race, see, e.g.,[41]).

The early literature on group polarisation, however, focussed on the extremity of group opinion. The historic point of departure for extremity research is the literature on “risky shifts” in decision-making [42] and the subsequent finding that groups would come to hold consensus views (beliefs or attitudes) that were more extreme than individuals’ pre-deliberation opinions (for early reviews see, e.g., [29,43]). For instance, Isenberg [43, p. 1141] states: “Group polarization is said to occur when an initial tendency of individual group members toward a given direction is enhanced following group discussion.”

Two dominant explanations for these dynamics emerged: social comparison processes (e.g., [44]) on the one hand, and “persuasive argumentation” [45] on the other. The social comparison explanation assumes that humans are motivated both to perceive and to present themselves in a socially desirable light (for agent-based models thereof see e.g., [46]). As a result, they continually monitor how others present themselves, and adjust their own self-presentation accordingly. Social comparison processes may give rise to extremity for two reasons. First, individuals may initially under-estimate the ‘true norm’ in the group (“pluralistic ignorance”) and then, subsequently, more readily reveal their own true beliefs, thus shifting the group average. Or, second, individuals may adapt their opinions due to “bandwagon” effects (see [43]). By contrast, the persuasive argumentation explanation assumes that individuals’ positions on an issue derive from the arguments for and against that they can recall when formulating their own position. Group discussion causes shifts because it exposes individuals to persuasive arguments that favor the direction in which opinion then polarises.

Needless to say, the two explanations are not mutually exclusive (despite attempts to empirically distinguish predictions in experimental contexts, see e.g., [45] and may each occur on some occasions or even occur together.

Polarisation as a move toward opinion extremity has since been a central topic in both psychology and political science, studied with a wide variety of methodologies from lab-based studies [47], to citizen debates [48], deliberative polling [49] and ‘citizens’ juries’ [50]. Considerable variation in magnitude of effects has been found: variation both by study (large effects, e.g., [49,50]; small effects, [51]), by topic of discourse, by aggregate level or individual [48,52]), and by measure of attitude (e.g., self-report or direct observation, [52]).

It is a virtue of persuasive argumentation accounts of shifts to extremity that (unlike social comparison theory) they provide a good account of when groups are expected to move to extremes and when not, as a function of initial attitudes and the type of information group members are likely to be able to put forward as a result ([28,43]). The ‘exchange of new information’ assumed by the persuasive argumentation theory renders shifts to extremity potentially rational as group members are simply responding to new information. Nevertheless, polarisation has often been seen as carrying at least a whiff of bias, in particular in political science, because groups are likely to include at least some diversity of opinion, and it would consequently seem that at least some of these opinions must be being ignored if beliefs overall become (uni-directionally) more extreme. In keeping with this suspicion of bias are some of the moderators, in particular, the fact that making group membership more salient enhances polarisation [53].

The intuitive charges of irrationality are brought to a head in Lord et al.’s seminal [25] study on polarisation as a process of belief divergence already mentioned above. Here, participants were presented with mixed evidence on the effectiveness of capital punishment as a deterrent of crime. Each participant read two (experimenter designed) journal articles, one purporting to show effectiveness and the other purporting to show ineffectiveness. Participants rated the report that agreed with their prior opinion as “more convincing,” and more readily found flaws in the reports that went against it. Moreover, the effect of each report on participants’ subsequent beliefs was stronger when the report agreed with their prior self-assessment as proponents or opponents of capital punishment. In other words, participants’ beliefs became more polarised by conflicting evidence that, if anything, should have made them less sure of their beliefs. This polarisation phenomenon, whereby the same (mixed) evidence leads people to reinforce their initial views, came to be known as “biased assimilation” and is viewed as one of the key pieces of evidence for motivated reasoning (see [54]).

Hence polarisation has come to be of theoretical concern to researchers focussed on understanding basic psychological processes of opinion, attitude, and belief formation (e.g., [55]), human rationality and bias [26], as well the democratic process and deliberative democracy (e.g., [37,56]), in addition to contemporary applied issues such as the climate and vaccine debates, the potential role of the internet and the advent of social media in promoting partisanship, conspiracy theories, and fake news.

In all of these contexts, it matters exactly why polarisation occurs. Yet past research has not always sought to distinguish clearly between rational and irrational, or epistemic and motivational accounts [28, chapter 2]. It may be that there are some general recommendations that can be made in applied contexts that may serve to reduce polarisation regardless of its exact nature; but, by and large, effective answers will need to understand more precisely why polarisation occurs.

Hence a view voiced increasingly in the political science literature is that an understanding of polarisation requires a clearer understanding of individual level mechanisms, including why some participants polarise their opinions while others moderate them (see, e.g., [48]).

Our modelling aides such understanding in multiple ways. First, it illustrates that polarisation does not imply motivated reasoning. This is important because the literature continues to view polarisation as stemming from cognitive-motivational biases (see e.g., [57]). Even “biased assimilation” is possible with fully rational agents. Whereas earlier commentators on the biased assimilation phenomenon had postulated a so-called “neutral evidence principle” for rational agents [58] whereby ‘neutral evidence’ such as the ‘mixed evidence’ in Lord et al.’s [25] study should not alter beliefs, more recent work within the formal framework of Bayesian probability has clarified that rational agents can respond differentially not just to ‘neutral evidence’ but to evidence more generally (see [26], for discussion). [59], for example, show how seemingly biased assimilation may occur in rational, Bayesian agents who assume different causal models. Olsson [9,26] identify how ‘biased assimilation’ may arise because perceptions of source reliability differ. These studies clarify that evidence cannot simply be stipulated to be ‘neutral’ by experimenters themselves, as it is the diagnostic impact that participants themselves assign that will guide their belief revision. It is entirely possible for rational agents, who have different background knowledge, to disagree about the diagnosticity of evidence reports; as a consequence, they will inevitably differ also in how much receipt of that evidence changes their beliefs.

Furthermore, the present modelling highlights that discrepant perceptions of evidential value will be prevalent in everyday life, because they stem from a fundamental epistemic problem: evidence tends not to come ready labelled with its diagnostic value. Diagnostic value is something that itself has to be learned, estimated or inferred. Or to put this in technical terms: even fully rational agents will have figure out what the likelihoods are. The modelling framework of Olsson [8] used in this paper puts this problem center-stage, allowing, for the first time, examination of the consequences of this central challenge that humans face as cognitive agents.

The updating agents modelled here implement, in a rational actor model, a strategy that humans use to estimate the reliability of testimonial evidence, see [7,11]. The agents adopt this strategy in order to enhance the accuracy of their beliefs, and, under certain conditions (see Fig 4), this strategy is objectively accuracy enhancing. These agents do not pursue the strategy for affective reasons–indeed they have no affect and they are incapable of motivated reasoning (for recent work on affective polarisation, see e.g., [60]). Yet, their behavior can *look like* motivated reasoning, including the possibility of backfire or boomerang effects where agents view evidence for a proposition as evidence against.

Vis a vis the literature on motivated reasoning (see [54]), this makes clear that attributing motivated reasoning requires evidence above and beyond differential responding to evidence that is or is not concordant with prior beliefs–ideally, such evidence should include direct evidence of affect. Biased assimilation does not reliably indicate the presence of motivated reasoning. And the mere presence of polarisation as a property of belief distributions within a society is even less diagnostic: both extreme beliefs, and split societies were shown to be a natural consequence of the update strategy in our simulations, which means accuracy motivated attempts to gauge the reliability of our sources, rather than affective bias, can drive these phenomena. But it was also seen in networked, fixed-trust agents. All of this supports the view that polarisation can come about through rational means (see also [28,43]), though motivational and/or social identity and comparison processes may, of course, make additional unique contributions in real world contexts.

Olsson [8] had already shown that polarised societies can emerge in collectives of updating agents and further work using his model [61] extended that work in larger simulations. Crucially, our results go beyond those initial findings by identifying a clear causal role of the update strategy through the comparison with the fixed-trust manipulation. This brings into focus the essential role of trust in the context of societal polarisation, not just in the sense of generalised institutional trust (e.g., [62]) but as a core component of any belief revision. Understanding the dynamics of credibility will be necessary to fully understand the impact of information, including misinformation and disinformation on social media (see also, e.g., [63–66]).

While our simulations thus contribute to the understanding of individual level mechanisms underlying polarisation, they also make clear that focus on this level alone is not enough. The clear network effects we observe indicate that it is equally important that individual belief revision is not considered in isolation (on the importance of network structure for studying polarisation, see already [67]). Being part of a social network fosters polarisation in our simulations and increasing the size of that network increases the rate at which societal polarisation is observed. This is true even for the fixed-trust agents in our simulations. This finding is important because it makes clear that differential weighting of evidence (whether rational or motivated) is not required to generate polarisation (contra the claims of both [61] and [68]), and differential information selection is not required either. While the evidential history of individual fixed-trust agents in our simulated world varies (different agents will see slightly different sequences of evidence from the world, and the evidence they receive via testimony will vary as a function of their neighbours), that variation is entirely stochastic: there is no sense in which the fixed-trust agents of our simulations intentionally or inadvertently expose themselves preferentially to belief-congruent evidence. Yet such selective exposure, either through self-curated news (see e.g., [37]), or algorithmic provision (“filter bubbles”, [69]) has been seen as a key driver, if not *the* driver, in the putative increase of polarisation through the rise of the internet. We next detail that research, and the implications of our findings for it.

#### Polarisation and the internet

While much of the early reception of the internet enthusiastically welcomed its likely effect on public and political discourse, e.g., [70], there has recently been mounting concern about potential negative impacts of the advent of online social media (e.g., [71]). This concern is fuelled by the fact that ever larger proportions of citizens engage in civic or political activities on the internet (in case of the U.S. over 50% for 2019, [72]) and politicians themselves are becoming ever more active on social media, thus transforming the political process.

In particular, there are increased concerns that social media might polarise politics (e.g., [37,73–77]). Some of these concerns stem from the changes in style and social impact brought about by online communication (see [78] and references therein), in particular anonymous online communication: these range from the negative but comparatively harmless effects seen in early studies (e.g., [79]) to the transgressive trolling of contemporary online culture wars (see e.g., [80]). However, the main concern with respect polarisation has arguably been homophily ([81]): “Similarity breeds connection” so that social networks see people linking to similar others (see also [82,83] for early work on homophily). This then may give rise to echo chamber effects. It is these echo chambers that are then viewed as the basis for further polarisation as belief congruent messages amplify extant beliefs and the failure to encounter opposing arguments makes pull back impossible.

Traditional media exposed people to greater diversity of opinion than they typically encountered in their social contacts (see, [84]); replacing traditional media with information from self-selected online sources may consequently radically alter the diversity of opinion encountered. Given that there is evidence that exposure to congruent views on the internet is associated both with the adoption of extreme positions and polarised political stances [85], and–conversely—it has been found that an individual’s network heterogeneity can increase their tolerance and understanding of other’s views (e.g., [86]), it is understandable that selective exposure is a potential cause for concern. These concerns are only fuelled further by the fact that algorithmic recommendations may amplify exposure to belief-congruent material, not just in the context of online information search [69], but specifically also in the context of social media, for example, through Facebook’s newsfeed algorithm (see e.g., [87]).

As a result, considerable amounts of research have focussed on establishing the extent to which social media actually do give rise to echo chambers. This work has examined both social media networks for political “elites” such as parliamentarians (see e.g., [74,88,89]) and the general public [85]. While there is evidence for echo chambers in political blogs [90] and microblogging on Twitter (e.g., [85,91,92]), others studies have indicated that the prevalence of echo chambers may have been overstated for Facebook [93], Twitter (e.g., [94–96]), for internet chatrooms [97], and for online news consumption [98]. In each case, users have been found to be subject to inadvertent exposure [99] to opposing views. In the words of Barbera et al. [96]: “homophilic tendencies in online interaction do not imply that information about current events is necessarily constrained by the walls of the echo chamber” (p. 9).

As the prevalence of “echo chambers” varies not only by social media platform/type and specific topic (see e.g., [94,97], but also type of user (e.g., varying by political interest, [10] or political ideology, [73,75]), there may not be a ‘general’ answer to the question of how prevalent echo chambers are, nor to the question of how influential they have become in fostering extreme views. Furthermore, studies of echo chambers face significant methodological problems in terms of sampling (e.g., [100,101]) and in terms of the breadth of information considered, providing opportunities for other methodologies such as user surveys on heterogeneity (see e.g., [97,102]). In particular, it is arguably necessary to consider not just one particular ‘network’ (say, retweets) but multiple concurrent layers [75,103,104], as well as the wider real-world social networks of the social media user [74] and the wider array of news media a user may be choosing from [105]. Dubois and Blank [105], in particular, argue that the value of empirical studies looking at a single medium is limited, given that, in their data, young people (18–34) have accounts on five media on average and the results of their study suggest that, at least, those who are politically interested avoid echo chambers in their overall ‘media diets’.

The theoretical focus on echo chambers as a root cause of polarisation has been driven by the idea that exposure to diverse views would decrease extreme views as others’ arguments are assimilated (e.g., [37]). However, research on internet mediated communication has also questioned whether the online exposure to opposing beliefs necessarily decreases extremity, or whether it might, in fact, fuel it (e.g., [95,103]) through “backfire effects” ([104,106–108]; but see also [109]) based on counterarguing [45], motivated reasoning [54], or social identification [44].

For example, [110] conducted a large, online field experiment in which participants were offered financial incentives to follow a Twitter bot for 1 month. This bot systematically exposed them to messages from those with opposing U.S. political ideologies (e.g., elected officials, opinion leaders, media organizations, and nonprofit groups). Worryingly, Republicans who followed a liberal Twitter bot became substantially more conservative, while Democrats exhibited slight (non-significant) increases in liberal attitudes after following the conservative Twitter bot.

Likewise, Wojcieszak [74], examining online white nationalist fora in the wider context of users other (non-internet based) social contexts, found evidence that both like-minded and dissimilar offline social ties serve to exacerbate extremism. Resistance to persuasion by those dissimilar ties is fuelled by the fact that users jointly engage in critical analysis, deconstruction and counter-arguing of the outgroup position. In fact, the particular neo-Nazi fora examined explicitly seek to “teach debating skills, inform how to use these skills during offline interactions, outline oppositional views and provide arguments to rebut those views” [74, p. 648].

Studies such as these provide essential insights, but the picture they present will nevertheless remain incomplete. Analyses such as those of [110] or [74] are focussed on the consequences of direct links, that means, in network parlance, between neighbouring nodes. Undoubtedly the internet has affected direct links in that it allows people to exchange views who might otherwise never have met giving rise to greater social network heterogeneity (see e.g., [111]), and media such as newspaper online comments fora have provided people with new (weak) ties. But profound changes lurk even where things seem to have stayed the same: the number of close contacts people have seems not to have changed through Facebook once demographic differences are controlled for [112], but the *overall structure* of the network in which those contacts are embedded has. Increasing that network to the point of globally “connecting everyone” has been part of Mark Zuckerberg and Facebook’s declared goal for decades (see e.g., https://www.facebook.com/zuck/posts/10154944663901634) and attempts to connect the remaining 4 billion inhabitants of the planet to its services are actively being pursued through the organization Facebook Connectivity (cf., [87]).

Our study shows effects of the scale of that network as a whole and how that scale interacts with our everyday mechanisms for estimating source reliability. Such systemic effects will be missed entirely by purely neighbourhood-based analyses. Modelling thus seems essential to identifying such effects which can eventually be taken back to the analysis of empirical networks (see e.g., [112]) and validated there.

#### From climate change to conspiracy theories

The potential role of online social networks in fostering polarisation is an issue not just with respect to politics and public debate in general, but also with respect to science and science communication.

Much of the research on people’s responses to climate science has focussed on the mediating impact of individual’s ‘worldviews’ in how climate communications are received [113,114]. These may give rise to differential perceptions and processing of messages via motivated reasoning and/or social identity concerns, with the consequence that communications about scientific findings fail to depolarise. Additionally, however, some research has posited underlying personality variables that may influence both political affiliation and response to value inconsistent information (e.g., [115]; but see [62]). On such a view, those holding opposing views on anthropogenic climate change differ not (just) as a result of differences in the information they have been exposed to, but in their psychological make-up.

Those same explanatory strands are found in research on ‘conspiracy theories’. In this research, one finds attempts to explain ‘conspiracist’ thinking in motivational terms (e.g., [116,117]). One finds also a growing literature that has sought to understand conspiracy theories by identifying individual differences that make people susceptible such as a need for cognitive closure, perceived lack of power, or propensity for illusory pattern perception (see e.g., [118–122]).

Finally, the topics of science-denial and conspiracy theory intersect directly in the context of the anti-vaccination movement (see e.g., [92,123], where attempts to counter vaccination myths have seen evidence of ‘backfire effects” ([124,125]; but see also [126]).

In all of these contexts, empirical research on individual differences seems both necessary and informative. However, it seems vital to not overlook systems level variables, in particular people’s information networks. This in turn draws attention to the changes in those networks that have been brought about through the rise of the internet: anti-vaccine messages, for example, have been found to be more widespread on the internet than in other media (see e.g., [127]) and are supported by “rumor communities” [128]; online fora such as reader comments have been examined with respect to debate about climate change (e.g., [129,130]), and the internet remains a rich source of conspiracy theory (e.g., [131]).

Empirical investigations such as those of [132] have sought to probe (de)polarisation on climate science in purpose-built online groups. [133] found evidence of ‘echo chambers’ in real-world U.S. climate policy networks. More generally, [134] have provided experimental evidence of effects of network structure on rumor spread and polarisation.

For statements of fact, both a claim and its opposite cannot both be true. Hence the network focussed literature on polarisation in the context of both science and conspiracy overlaps with the growing literature on the spread of misinformation (e.g., [135,136]) and attempts to counter it (e.g., [137]). This focus on information networks complements studies of individual level characteristics governing susceptibility to misinformation (see e.g., [138–141]).

Our modelling has multiple implications for these literatures: it shows how even purely accuracy motivated cognitive agents may end up with a “crippled epistemology” [142] that leaves them detached from objective reality, through no fault of their own, solely through an ‘unlucky’ evidential history. This is true even for agents that are uniformly trusting of their sources; but it is magnified when expectation-based updating of source reliability is added to the equation. Those ‘conspiracy theorists’ in our simulated worlds who strongly end up believing a falsehood, end up in this position through sheer bad luck. In everyday life, it is tempting to view those with radically opposing views of facts to be subject to bias or deficient reasoning. By contrast, our simulations demonstrate how the basic mechanisms of expectation-based updating and social communication, mechanisms that are genuinely accuracy enhancing (see Fig 6), may conspire to lead beliefs astray.

Moreover, expectation-based updating fits with past research on misinformation such as the finding that conspiracies ‘go together’. Specifically, people who believe in one conspiracy, are also more likely to believe in others. This seems plausible once it is realised that beliefs about message content and message source will interact. Once a conspiracy promoting source seems credible, other offerings from that source will also have an effect. Conversely, once conspiracy seems plausible, the perceived reliability of conspiracy-promoting sources will increase. Hence, expectation-based updating suggests an epistemic, non-motivational basis, for such findings, which fits both with the moderating role of trust observed on empirical work on seemingly motivated cognition in conspiracy adherents (e.g., [116]) and findings of lower levels of interpersonal trust in adherents of conspiracy theories (see e.g., [143]).

In other words, there are grounds for a focus not on individual differences as inherent personality variables, but rather on people’s experiential history, wherein arguments and perceived source reliability evolve dynamically over time. Further support for the role of experiential history (as opposed to intrinsic personality traits) can be found from the links between conspiracy ideation and internet use [117] as well as individual case studies ([144].

At the same time, the clear effects of network size in our simulations underscore the importance of supra-individual, systems level variables. Specifically, those results suggest that the prevalence of conspiracy theories and misinformation can rise simply because of changes to the effective size of our everyday information networks in ways that the early enthusiastic reception of the internet and social media could not have anticipated. The fact, finally, that individual agent strategies (expectation-based update vs. fixed trust) interact with network characteristics in our simulations, suggests that agent-based simulations that allow one to isolate and explore the impact of putative causal variables provide an important, and arguably necessary, additional methodological tool in order to gain a complete understanding of the complex real-world dynamics involved in climate beliefs, anti-vaccination movements or conspiracy theories.

Hence it is useful, finally, to situate the present work in the wider context of current modelling efforts aimed at understanding belief and opinion dynamics generally, and polarisation specifically.

#### Other models

Our simulations establish the causal role of a basic strategy for gauging source reliability, expectation-based updating [7], in generating polarisation. This strategy, which is associated with accuracy gains, at least in some contexts (see Fig 6), is shown to have pernicious side-effects in social networks when external evidence is less reliable. Moreover, the polarising effects of this strategy are enhanced by network size, which, in and of itself, promotes polarisation.

In short, the modelling presented here identifies novel factors that fuel the emergence of polarisation within a population of initially entirely homogenous agents. By contrast, the vast majority of modelling work on polarisation to date has examined how polarisation in initially diverse populations may be maintained.

For example, there is an extensive body of research using models of repeated (weighted) belief averaging, such as the DeGroot [145] or Lehrer-Wagner [146] model. These models provide little insight into polarisation because beliefs in the DeGroot/Lehrer model will generally converge to a common value over time. Golub and Jackson [147,148] generalised the DeGroot model by introducing the notion of a ground truth into the model in order to explore the impact of network structure on accuracy. Hegselman and Krause [149] added weights reflecting differential ‘trust’ in other members of the collective so that agents only listen to others who are sufficiently close in their estimates. There has been much work on this class of models, involving interesting extensions ([150,151]; for a review, [152]). The presence of the source reliability threshold, which is central to this model class, means that network structure (who agents pay attention to) changes dynamically over time. However, the threshold value itself is arbitrary and unchanging. Hegselman and Krause’s interests lay in understanding the impact of such a threshold on when networks do and do not converge (see for extensive analysis, [153]). But agents in this framework must already start out with heterogeneous beliefs, otherwise polarisation will not emerge. A closely related type of “bounded confidence model” is the Deffuant and Weisbuch model (see e.g., [154]), used also by [155,156] to study polarization. [154] allows changing thresholds, but otherwise comments on Hegselman-Krause apply here as well.

Our modelling is also distinct from other work in that the majority of modelling concerned with opinion dynamics across social networks has pursued the issue from either a ‘social physics’ perspective [157], employing models based on Hopfield networks, or has modelled the spread of opinion using contagion models drawn initially from epidemiology (see e.g., [158] for an introduction). This includes modelling concerned specifically with polarisation (e.g., using Hopfield networks, e.g., [159]; and in a contagion framework, [160]). These frameworks model opinion spread without reference to a ‘ground truth’ in the simulated world that would allow one to gauge the accuracy of agents’ beliefs; nor do they attempt to identify more closely either what human’s ought to do or actually do.

By contrast, the naïve Bayesian agent of the Olsson model has a clear normative basis (see e.g., [2]), was advanced as a putative model of how humans should deal with testimony, and there is empirical evidence (e.g., [7,11]) that the general strategy of expectation-based update, which it implements in a Bayesian framework, is, in fact, a strategy that humans actually employ.

Other simulations have shown that optimal belief revision and information from the world are not enough to guarantee that rational agents converge on the truth: rational, Bayesian agents may be subject to information cascades (where agents fall in line with some, possibly erroneous, tendency, see e.g., [161,162] on information cascades in general, and e.g., [163], for a Bayesian model thereof). Likewise, [9,164] show initially heterogenous populations of Bayesian agents sustaining polarisation despite input from the world. [165] showed how societies who contain a proportion of stubborn agents who never change their beliefs will prevent the emergence of consensus in the *remaining* population of Bayesian agents who do revise. Finally, polarisation has been studied within the framework of the Bala and Goyal [166] model (see e.g., [20,167]), a model widely used within philosophy (see also, [168–171]). This model, too, involves Bayesian agents, but unlike the present simulations, examines the effects of information selection. Tensions between exploration and exploitation that arise in the context of information selection may also give rise to polarisation, even in entirely rational agents.

By identifying expectation-based updating, network size and their mutually reinforcing relationship as causal factors in the emergence of polarisation, the present work goes beyond these past studies by connecting polarisation to the fundamental challenge of knowledge acquisition in a social world: much, if not most, of what we believe to know, we know through the testimony of others, but the reliability of that testimony is something we have to judge. Our simulations highlight the scale of that challenge, the limitations of expectation-based updating as a strategy for estimating source reliability, and the extent to which the accuracy of our beliefs is determined by collective level properties such as network size that are beyond the reach of the individual cognitive agent. As such, they suggest that the accuracy of our beliefs is, for each and every one of us, less under our control than we may wish to think.

### Conclusions

Concern has spread about the impact of technological developments such as Twitter and Facebook and their impact on the beliefs of their users. While earlier discussions argued strongly that these new technologies were ‘just another platform’, no different, in principle to a postal system, more discussion has started to accept the idea that something important might have changed. However, it presently remains poorly understood what that might be. Much of the focus has been on willful attempts at manipulation or deceit, whether through economically motivated “fake news” or targeted, politically motivated intervention [172]. While important in their own right, these angles miss what seem to be more fundamental characteristics. The simulations reported in this paper indicate clearly that *scale matters*. Increasing the effective size of one’s social network, in and of itself, has consequences for belief polarisation. Crucially, increasing the size of people’s communication networks and increasing the frequency of communication is not merely a side effect of Facebook or Twitter, it is the very point of those projects. This raises doubts that there are comparatively straightforward ‘fixes’ to these platforms that will mitigate polarisation and its adverse societal consequences.

At the same time, the simulations presented make clear that information integrity (which is compromised by “fake news” or deceit) matters strongly in this context. Both accuracy and polarisation are strongly affected by the reliability of the information fed into the network from external sources. Where information entering the network is entirely consistent, there is no basis for beliefs to diverge.

Finally, expectation-based updating itself gives rise to polarisation. Such a strategy weights testimony by the extent to which it is congruent with one’s present belief about the claim in question; consequently it gives rise to a kind of ‘confirmation bias’, whereby belief-congruent evidence becomes amplified, and incongruent evidence down-weighted (e.g., [173]). This matters because experimental evidence suggests that people actually use such a strategy [7,11]. This strategy, as implemented in our simulations, reflects ***only*** an accuracy motivation (cf., e.g., [37]). The agents in our simulations are *doing their best* to form accurate beliefs about the world. They do not suffer from other motivational biases (see [26]), tendencies to avoid seeking out belief-conflicting evidence in the first place (e.g., “echo chambers”, see [90,100,136]), possibly aggrevated by filter bubbles [93] or the fact that lies, as more surprising, may travel further and penetrate beliefs more deeply [174]. In other words, there are likely many additional factors at play in the real world which will only make the problems worse.

For anyone concerned about belief accuracy and polarisation in the age of social media, the problems revealed by these simulations seem deep and structural, and unlikely to be remedied simply by improving users’ internet ‘savvy’ (see e.g., [175]). Communication across networks, in and of itself, fosters polarisation. It does so, because communication across a network amplifies evidence entering the network from the world, effectively giving rise to ‘double-counting’ (and, again, all of this happens before one factors in intentionally ampliative effects of Facebook and Twitter who actively promote ‘trending’ messages or content, thus further increasing ‘double counting’). This in turn interacts negatively with what seems (at least initially) like a natural, rational, strategy for gauging the reliability of others in context where we must rely on their evidence yet their accuracy/reliability is not known–a strategy which seems to be part of our basic psychological make-up [7]. Together, both factors multiply each other’s downsides. Yet communication networks are indispensable to humans as a species. Without testimonial evidence, there would be no culture, no science, no technology. It is thus a pressing practical challenge to get the balance (and scale!) of social networks right; we will not be able to do so without factoring in the fundamental mechanisms human beings have for gauging the reliability of their sources and the unanticipated consequences these can have in multi-agent settings.

## Supporting information

S1 Fig (TIFF)

S2 Fig (TIFF)

S3 Fig (TIFF)

S4 Fig (TIFF)

S5 Fig (TIF)

S6 Fig (TIFF)

S7 Fig (TIF)

S8 Fig (TIF)

S9 Fig (TIF)

S1 File (DOCX)
