# Supplementary figures and images for: Knowledge through social networks: Accuracy, error, and polarisation
Source: PLoS One. 2024 Jan 3;19(1):e0294815. doi: 10.1371/journal.pone.0294815 (PMC10763946; doi:10.1371/journal.pone.0294815)

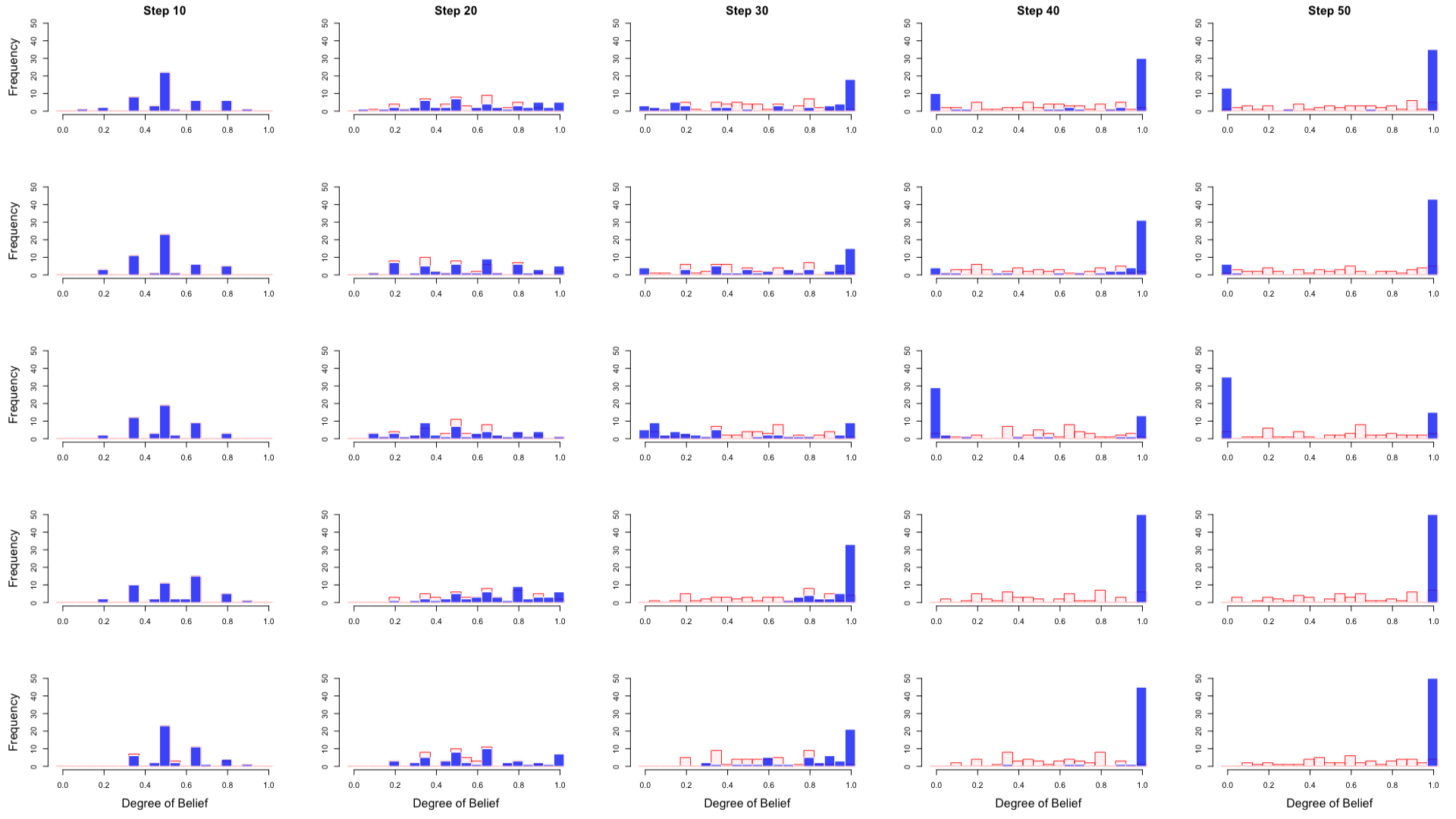

Supplement: S1 Fig — (TIFF) [file pone.0294815.s001.tiff]

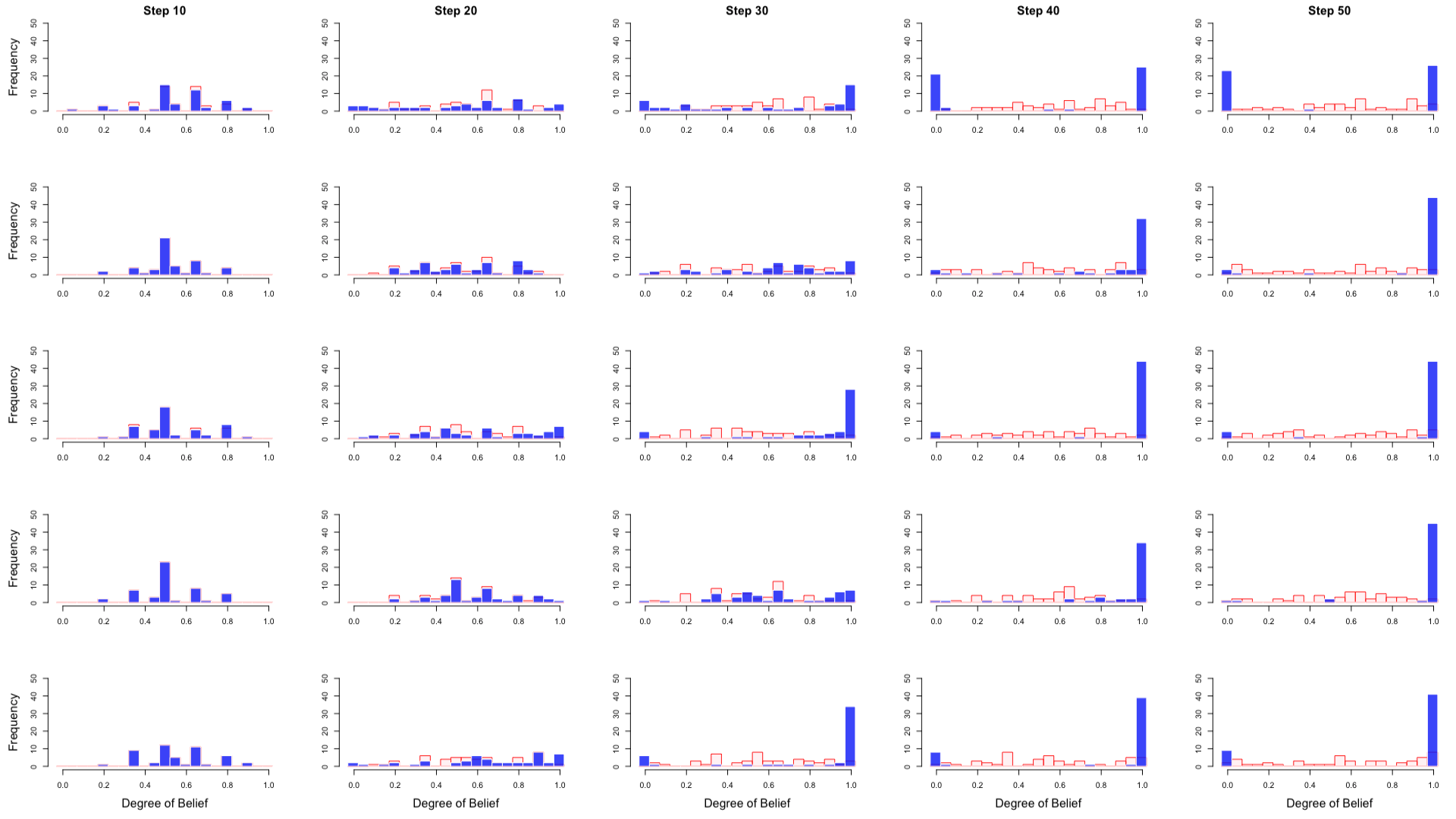

Supplement: S2 Fig — (TIFF) [file pone.0294815.s002.tiff]

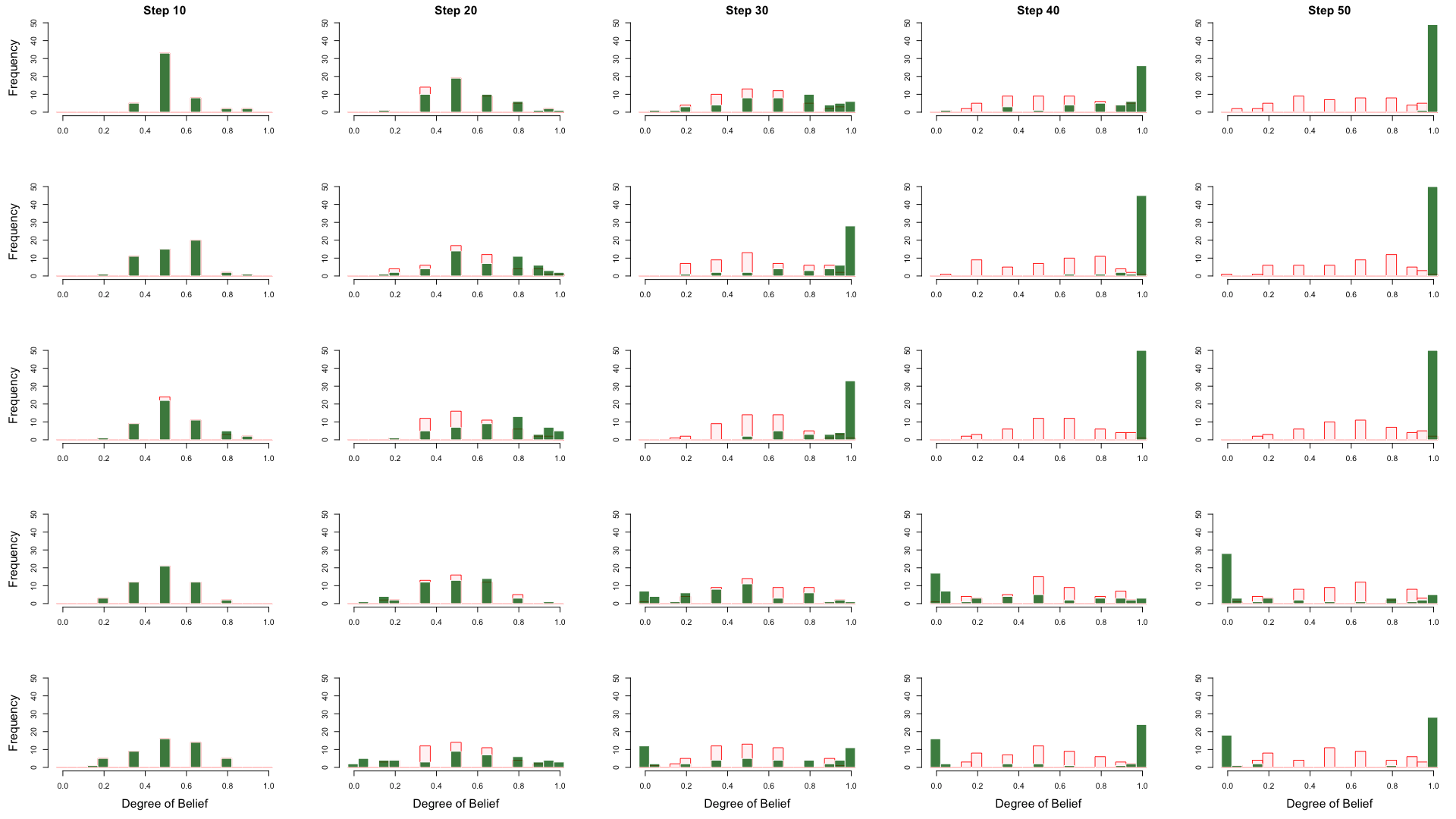

Supplement: S3 Fig — (TIFF) [file pone.0294815.s003.tiff]

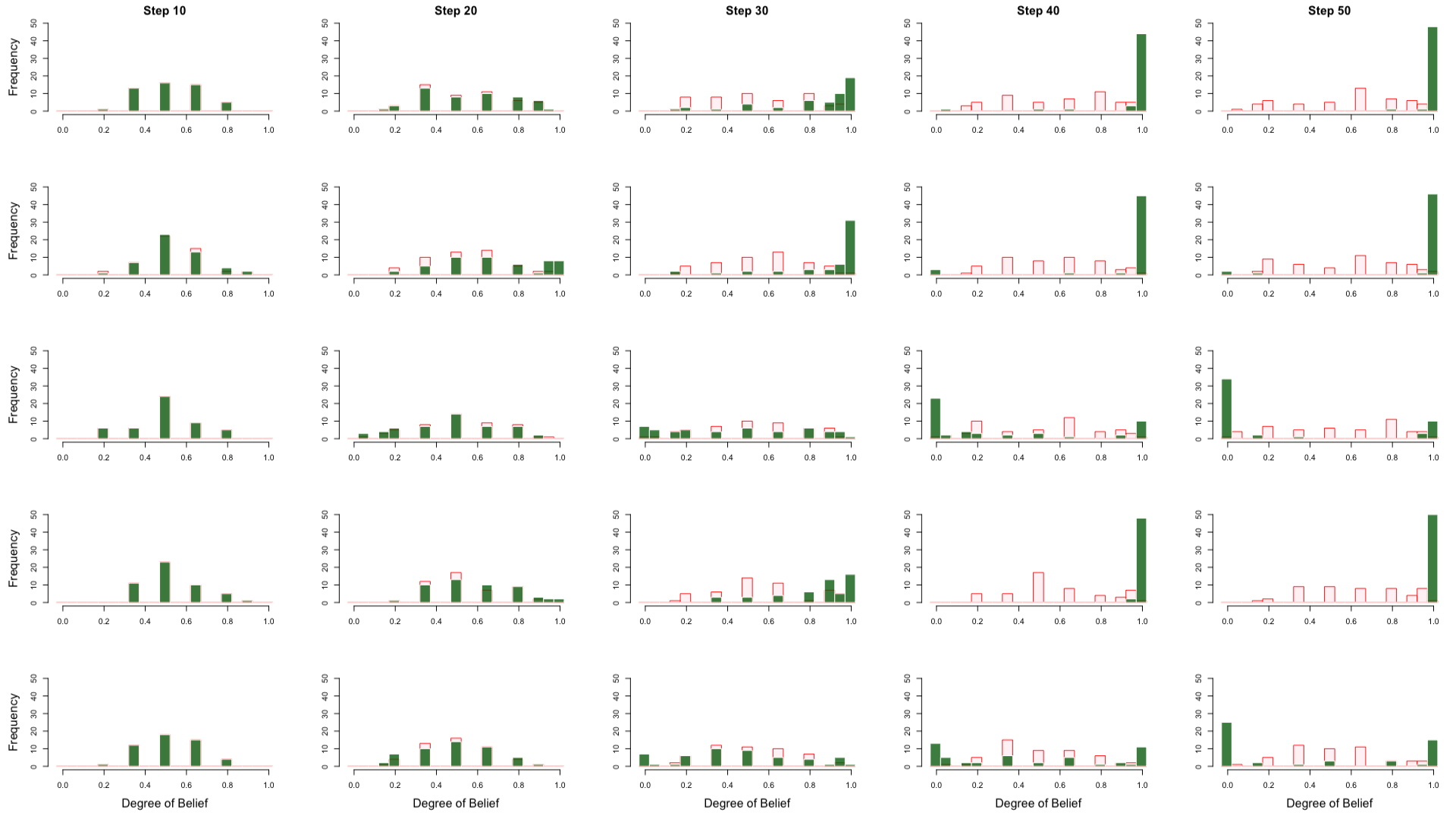

Supplement: S4 Fig — (TIFF) [file pone.0294815.s004.tiff]

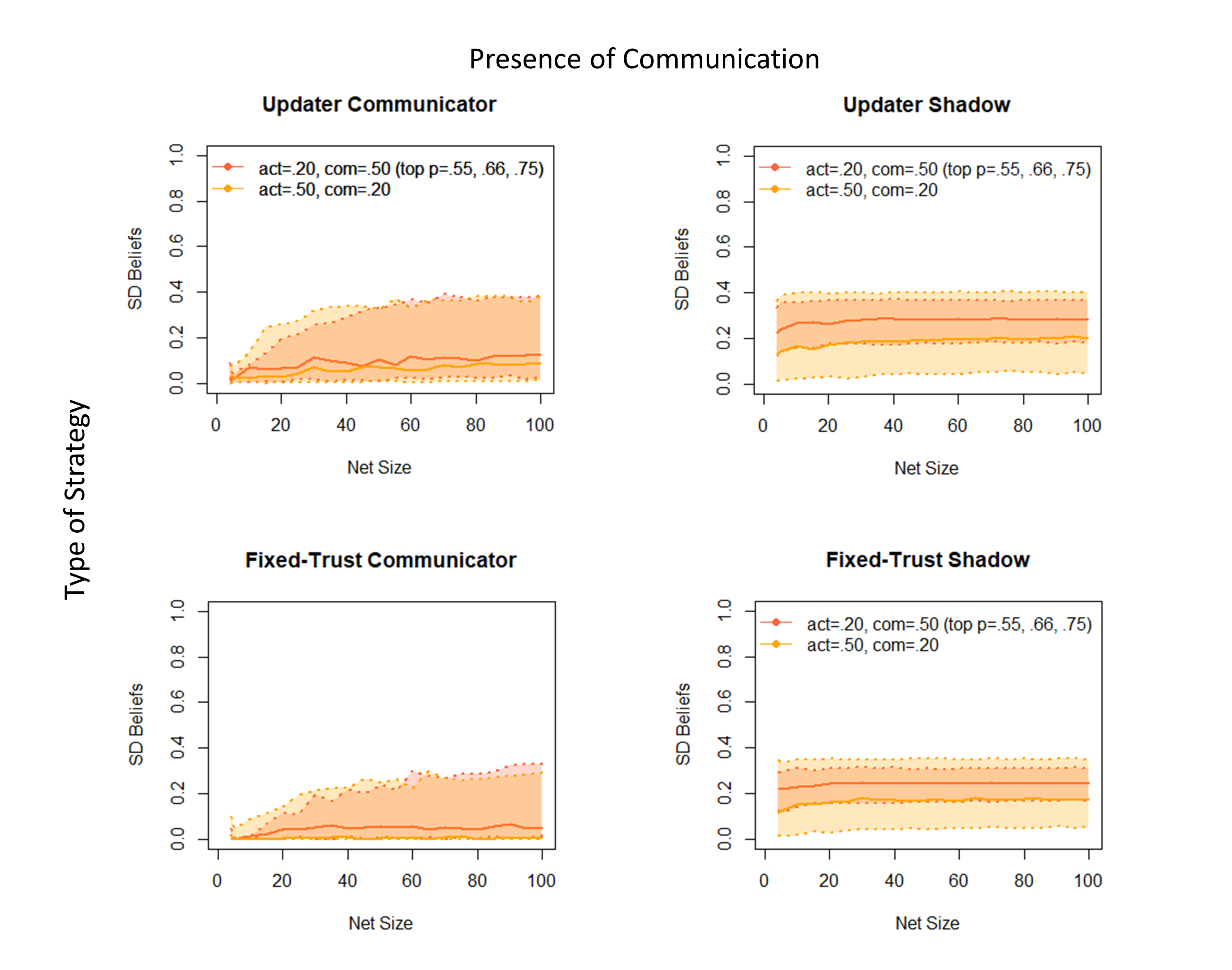

Supplement: S5 Fig — (TIF) [file pone.0294815.s005.TIF]

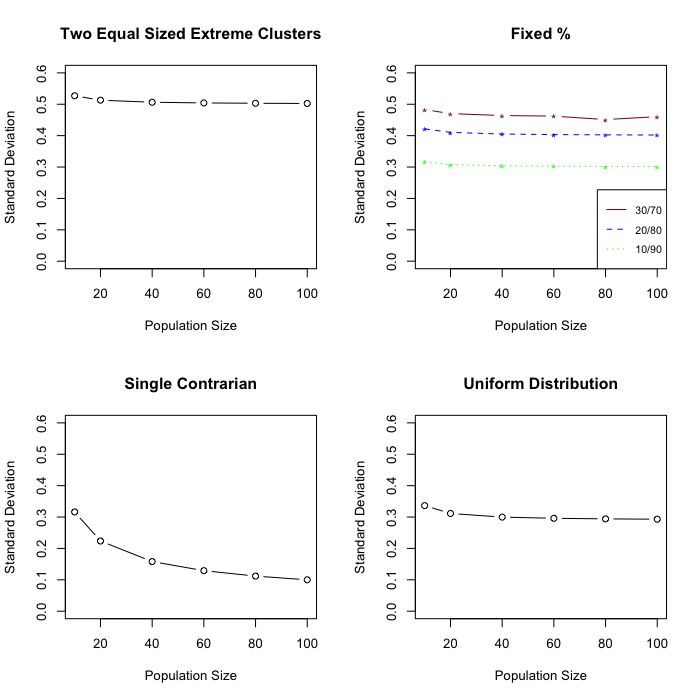

Supplement: S6 Fig — (TIFF) [file pone.0294815.s006.tiff]

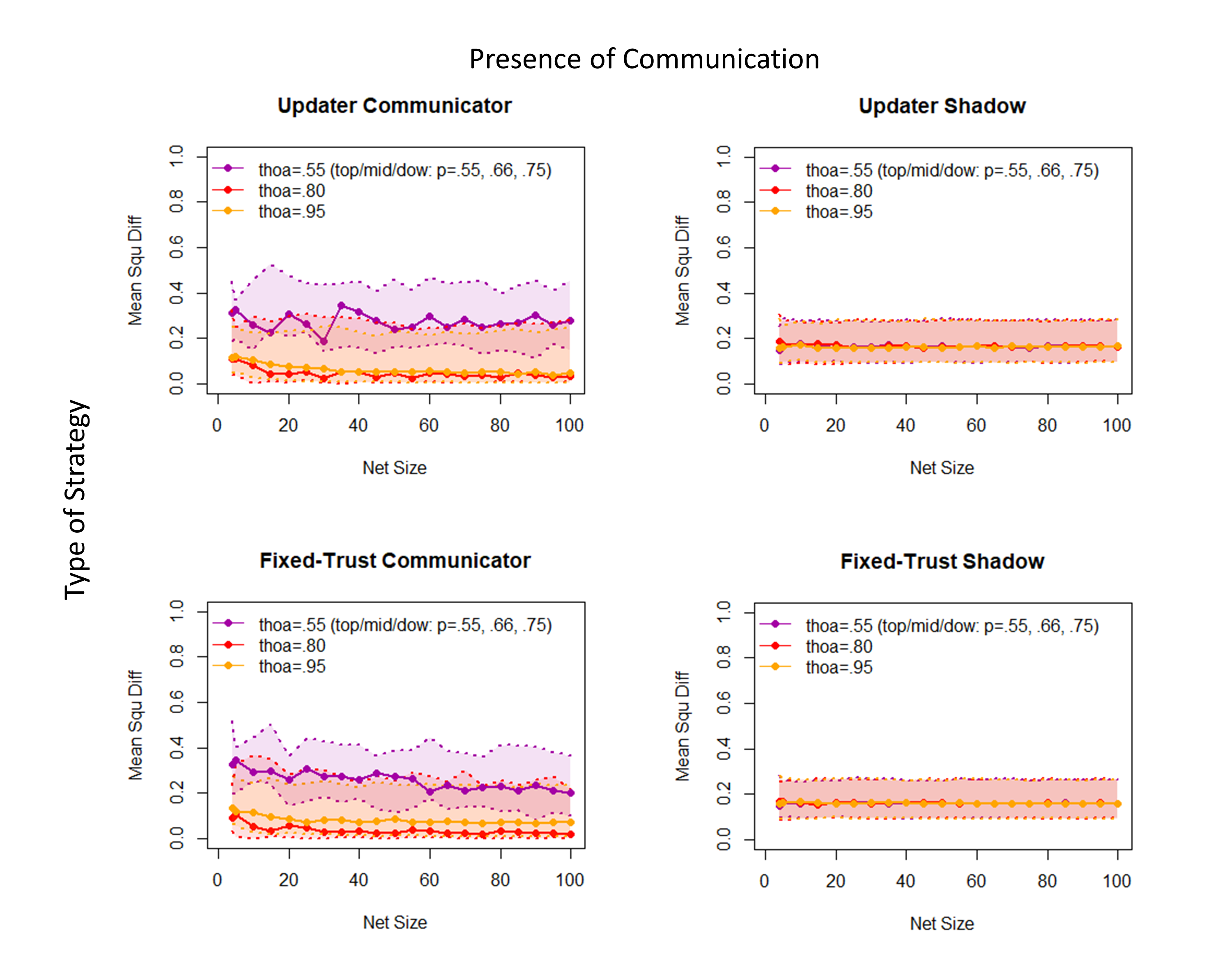

Supplement: S7 Fig — (TIF) [file pone.0294815.s007.TIF]

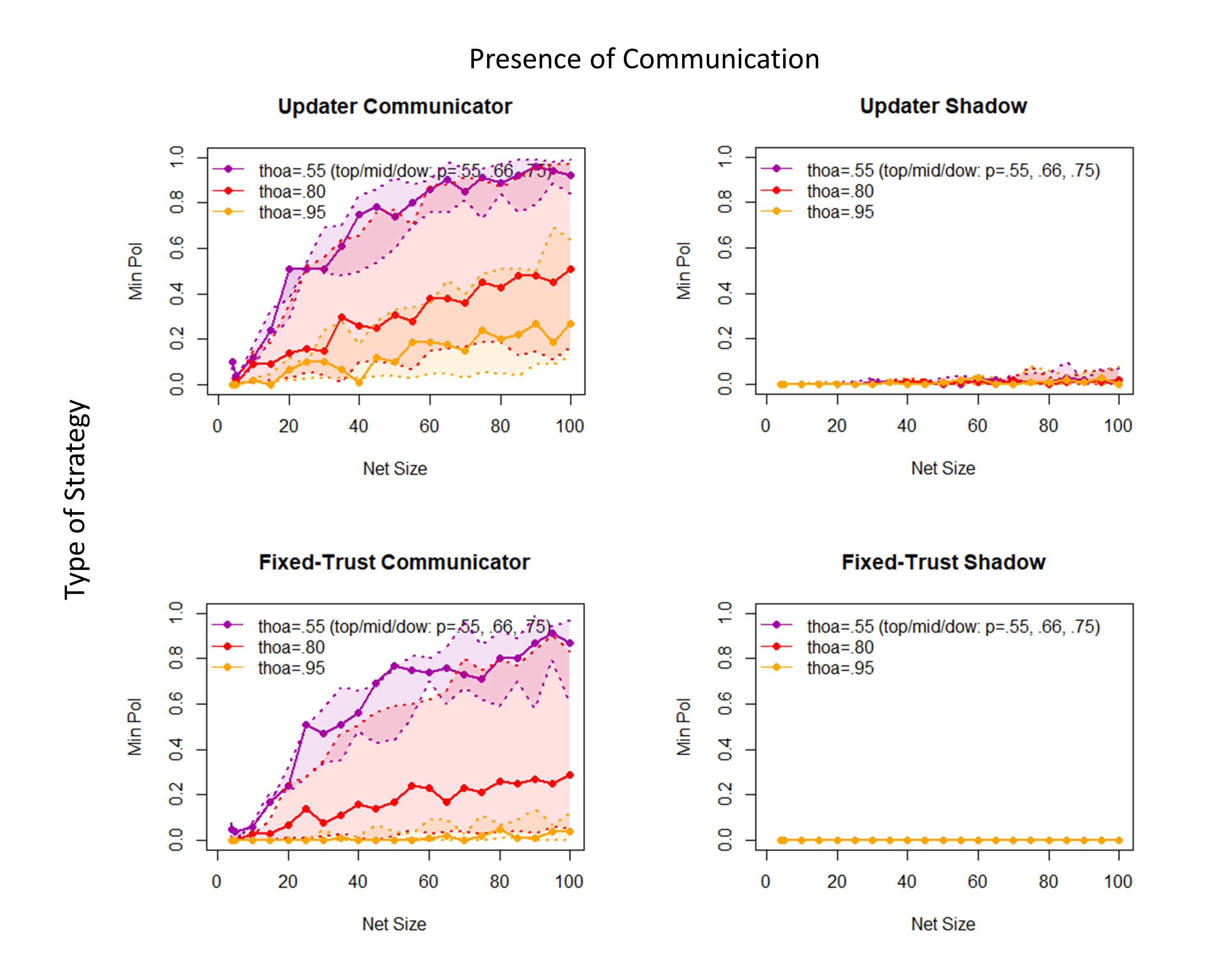

Supplement: S8 Fig — (TIF) [file pone.0294815.s008.TIF]

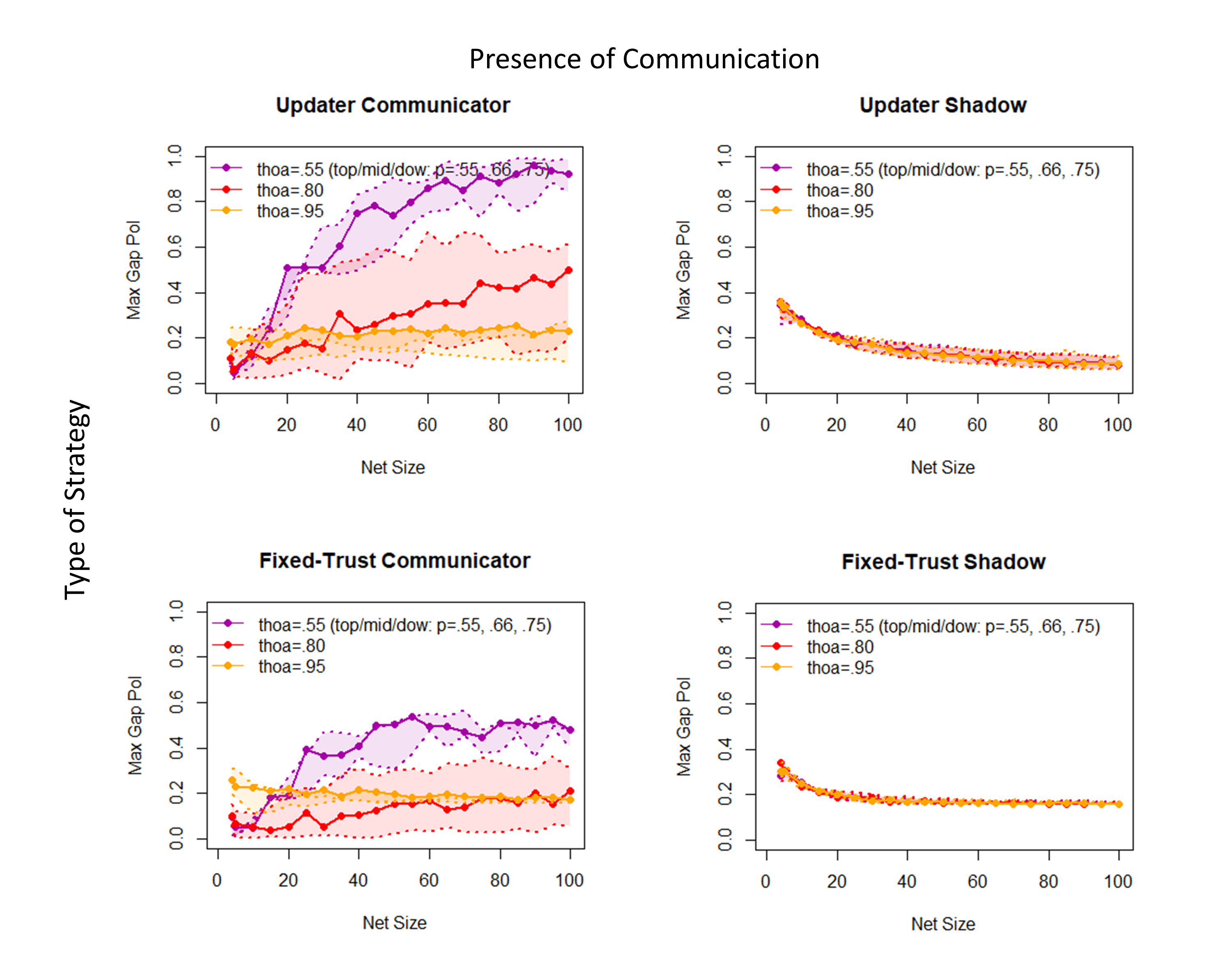

Supplement: S9 Fig — (TIF) [file pone.0294815.s009.TIF]
