# Supplementary material for: Knowledge through social networks: Accuracy, error, and polarisation
Source: PLoS One. 2024 Jan 3;19(1):e0294815. doi: 10.1371/journal.pone.0294815 (PMC10763946; doi:10.1371/journal.pone.0294815)
Supplement: S1 File — (DOCX) [file pone.0294815.s010.docx]

# Supplemental Materials

## Supp. Mat. A: Model Details

The model describes a community of investigators receiving evidence on a hypothesis of interest and exchange their beliefs, treating both each other and themselves as partially reliable sources of information. The agents send dichotomized reports to their neighbors on a network if their credence exceeds a threshold of assertion, and thereby try to learn both the truth of the hypothesis and the actual reliability of all perceived sources by a variant on Bayesian updating.

### State Variables, Entities and States

The model contains agents situated on a communication network, represented by the following state variables:

| Degree of Belief | An agent’s credence in a binary hypothesis. |
| --- | --- |
| Trust | Agents maintain a list of trust distributions for all sources they receive evidence from. |
| Links | Every agent has 2k links to other agents. |

### Processes and Scheduling

The model encompasses two main processes:

1. Data Collection

2. Communication

At each time step, first all agents engage with some probability in data collection. All agents possibly acquire a datum in random order, as determined by Netlogo’s scheduling algorithm (n.b., the within-process sequencing is functionally irrelevant since there is no interaction during data collection). When data collection is finished for that time step, agents engage in communication with their network neighbors. The agents become active in randomized order as defined by NetLogo‘s **ask** command. Each agent then communicates (conditional on several variables detailed in the subprocess section) to its neighbors. Agents immediately update belief and trust upon receiving a report. Hence, the overall schedule of communication is implemented as randomized sequential updating.

### Design Concepts

| Basic Principles | Agents engage in simultaneous Bayesian updating of belief and trust. |
| --- | --- |
| Emergence | Complex patterns of collective belief and trust (and distrust) relationships emerge, among other things polarisation on the social and convergence on the individual level. |
| Adaption | Agents follow a constant learning strategy, though adapt their reliability estimates to parametrize learning. |
| Objectives | Agents try to learn the truth about a hypothesis of interest and the reliability of sources. |
| Learning | At the core, agents try to simultaneously learn about a hypothesis and the reliability of their various sources, including themselves. |
| Prediction | Agents do not engage in prediction to inform their actions. |
| Sensing | Agents are able to perceive one piece of evidence from the world per time step, and sense the reports of all their network neighbors. |
| Interaction | Agents interact merely by the exchange of reports. |
| Stochasticity | The world as a source of evidence provides information probabilistically to represent the possibility of measurement error. Furthermore, agents not necessarily take all possible actions each time step; their activity is regulated probabilistically, too, to model variation in the agents‘ activities. |
| Collectives | There are no explicit collective entities, but the agents are situated on a network. |
| Observation | The dynamics of evolving belief can be observed. For most numerical experiments, though, only the final trust and credence values of all agents are recorded, and the correct value of the hypothesis for that run. |

### Initialization

| Prior | Initial value for the agent’s degree of belief. Most often initialized as 0.5. |
| --- | --- |
| Alpha, beta | Parameters for all initial trust variables, initialized as beta distributions. Most often initialized as 2,1 resulting in a moderately trusting initial stance. |
| p | Actual generating probability for accurate information from the world. Varied in 0.05 steps from 0 to 1. |
| p-hypothesis | Baserate of the hypothesis being true. Varied from in 0.05 steps from 0 to 1. |
| Threshold of assertion ‘thoa’ | Minimum credence to send out reports; note that credence has to be either larger than the threshold value or smaller than 1-threshold. Commonly used values are 0.55, 0.7, 0.9. |
| Global-activity ‘p-activity’ | Probability for an agent to collect data during a time step. Common value 0.2. |
| p-communication | Probability for an agent to communicate when they otherwise are enabled to do so. |
| Fixed-trust | Likelihood used by agents who do not update their trust values. |
| N | Number of agents |
| k, rewiring probability | Parameters for small-world generation. 2k is the number of neighbors for every agent, rewiring-probability defines the probability for each link to be stochastically rewired. |

### Input Data

The model does not utilize empirical data.

### Submodels

*Small-world generation:* The generation of small-world networks follows the algorithm described by

Flache, A., & Mäs, M. (2015). Multi-Agenten-Modelle. In *Handbuch Modellbildung und Simulation in den Sozialwissenschaften* (pp. 491-514). Springer VS, Wiesbaden.

The following submodels are all described as they apply per agent; see the processes and scheduling section for the ordering of updates across agents.

*Data collection:* With probability global-activity (p-activity), the agent receives a piece of evidence, represented by 0 or 1. This value is determined by the actually true hypothesis (also represented by 0 and 1) and the generative probability p: with probability p, the agent receives a report reflecting the truth about the hypothesis, with 1-p the opposite instead. The agent then updates its degree of belief and trust for themselves according to equations 1 and 2.

*Communication:* If the agent’s degree of belief is larger than the threshold of assertion or smaller than 1-threshold, the agent sends to each of its neighbors reports with probability p-communication. The agent sends a report of 1 if their credence is larger than 0.5 and 0 if is smaller. For a threshold of assertion of 0.5, the behavior is undefined. Upon receiving reports, the neighbors update their degree of belief and trust in the focal agent according to equations 1 and 2.

The alternative fixed-trust agents use Equation 1 and replace the expectation of the trust distribution with a constant.

*Update*: Trust and belief are updated simultaneously for a single piece of evidence. Following Olsson (2011, 2013), the model assumes evidence symmetry: that is, $P\left( e | h \right)=P\left( \neg e | \neg h \right).$

As a consequence, only a single parameter *p*, representing the probability of the evidence given the hypothesis, is necessary to characterize the evidence and positive and negative test results are equally diagnostic, i.e., LHR+ = LHR–.

Belief update is guided by Bayes’ theorem and the Principal Principle, resulting in the expectation of the reliability distribution τ figuring as the likelihood *P(e|h).* This leads to the following equation for a positive report *e*, suggested by Olsson (2013):

$P_{t+1}\left( h \right)=P_{t}\left( h | e \right)=\frac{E\left[ \tau_{t} \right]*P_{t}\left( h \right)}{E\left[ \tau_{t} \right]*P_{t}\left( h \right)+\left( 1-E\left[ \tau_{t} \right] \right)*\left( 1-P_{t}\left( h \right) \right)}$(Equation 1)

where the trust is updated for a positive report according to

$\tau_{t+1}\left( h \right)=\tau_{t}\left( h | e \right)=\frac{x*P_{t}\left( h \right)+\left( 1-x \right)*\left( 1-P_{t}\left( h \right) \right)}{E\left[ \tau_{t} \right]*P_{t}\left( h \right)+\left( 1-E\left[ \tau_{t} \right] \right)*\left( 1-P_{t}\left( h \right) \right)}*\tau_{t}\left( x \right)$(Equation 2)

The Olsson model represents reliability as a probability distribution over possible reliabilities, with a density function ranging over the unit interval [0,1]. The update equation defines a pointwise update of the distribution, and x denotes the element of the domain of the density function for which the update is performed.

Note that, as a result of symmetry, the probability of particular sequences of *n* pieces of evidence is described by a binomial probability *B*(*n, P*(*e|h*))*.*

The probability of exactly *k* successes in *n* trials is,

$f\left( k;n,p \right)=P\left( X=k \right)=\left( \begin{aligned} n \\ k \end{aligned} \right)p^{k}\left( 1-p \right)^{n-k}$ (Equation 3)

For k = 0, 1, 2, …, n, where

$\left( \begin{aligned} n \\ k \end{aligned} \right)=\frac{n!}{k!\left( n-k \right)!}$ (Equation 4)

and the expected value (mean) is *np.*

Note also that the proportions of expected trials receiving *k* pieces of positive and *n*–*k* pieces of negative evidence are determined by the true underlying parameters (‘the world’), not the agent’s beliefs about those parameters.

Our re-implementation of the Olsson (2011) model follows the original in all mathematical detail. However, we added several additional features for analytic purposes.

1. In the original model, the hypothesis is always true by convention. Hahn et al. (2018) added the feature that, on a given run, the value of *h* is randomly drawn according to the base rate (‘p-hypothesis’, labelled p*_obj_* in the legends of our graphs) set for the model run. Analyses for which this is useful play no role in the current simulations, and that base rate is uniformly set to .6 in all reported simulations.
2. Hahn et al. (2018) also added the possibility of agents having a fixed trust value, that is not updated via Eq. 2. Hahn et al. (2018) added fixed-trust agents in order to serve as a comparison for the expectation-based updating strategy. Fixed trust agents are initialised with initial trust τ which is a point distribution. Its value is not changed.
3. We added, for the present work, ‘shadow agents’ These are a central analytic tool in our simulations. ‘Shadow agents’ are so labelled because they ‘shadow’ the networked agents. At network initialisation, each agent in the network is assigned a unique ‘shadow agent’. This agent is assigned the same initial values as the network agent: that is, the same prior for *h*, and the same trust distribution τ concerning the agent’s own inquiry, that is, evidence received by the agent ‘from the world’. Whenever a network agent receives a piece of evidence from the world, the agent’s shadow agent receives this evidence also and updates its belief using Eq. 1. Shadow agents do not participate in communication.

**Pseudo Code for the Central Algorithm.**

**NOTE: for-loops are to be read as traversing a list in some order. In the NetLogo implementation, this order is determined by the random scheduling of the ask agents command.**

**for all** agents *i* **do**

Initialize *P_i_(h)*

*copy P_i_(h) to shadow(i)*

**end for**

**for all** agents *i* **do**

**for all** agents *j* **do**

Initialize trust prior distribution of *i* in *j*, *τ_ij_(x)*

**end for**

**end for**

Initialize *network-neighbors* to a small-world network

Draw *H* from 0,1 according to the base rate

**for** *n* timesteps **do**

**for all** agents *i* **do**

**if** *random*(0,1) < p-activity **then**

Generate *e* according to the objective likelihood given *h*

Set *P_i_(h)* to *P_i_(h*|*e)* according to Eq. 1

Set *P_shadow(i)_(h*) to *P_shadow(i)_(h*|*e*) according to Eq. 1

Set *τ_ii_(x)* to *τ_ii_(x*|*e)* according to Eq. 2

Set *τ_shadow(i),shadow(i)_(x*) to *τ_shadow(i),shadow(i)_(x*|*e*) according to Eq. 2

**end if**

**end for**

**for all** agents *i* **do**

**if** *random*(0,1) < *p-communication* AND *P_i_(H)* > *thoa* OR *(*1 *– P_i_(H) > thoa* **then**

**if** *P_i_(H)* ≥ 0.5 **then**

Set *REP* to 1

**else**

Set **REP** to 0

**end if**

**for all neighbors j of *i* do**

Set *P_j_(h)* to *P_j_(h*|*REP)* according to Eq. 1

Set *τ_ji_(x)* to *τ_ji_(x*|*REP)* according to Eq. 2

**end for**

**end if**

**end for**

**end for**

**Fixed trust agents**

The adapted algorithm for fixed trust agents consequently runs as follows:

**for all** agents *i* **do**

Initialize *P_i_(h)*

*copy P_i_(h) to shadow(i)*

**end for**

**for all** agents *i* **do**

**for all** agents *j* **do**

Initialize trust of *i* in *j*, *τ_ij_(x)*

**end for**

**end for**

Initialize *network-neighbors* to a small-world network

Draw *H* from 0,1 according to the base rate

**for** *n* timesteps **do**

**for all** agents *i* **do**

**if** *random*(0,1) < p-activity **then**

Generate *e* according to the objective likelihood given *h*

Set *P_i_(h)* to *P_i_(h*|*e)* according to Eq. 1

Set *P_shadow(i)_(h*) to *P_shadow(i)_(h*|*e*) according to Eq. 1

**end if**

**end for**

**for all** agents *i* **do**

**if** *random*(0,1) < *p-communication* AND *P_i_(H)* > *thoa* OR *(*1 *– P_i_(H) > thoa* **then**

**if** *P_i_(H)* ≥ 0.5 **then**

Set *REP* to 1

**else**

Set **REP** to 0

**end if**

**for all neighbors j of *i* do**

Set *P_j_(h)* to *P_j_(h*|*REP)* according to Eq. 1

**end for**

**end if**

**end for**

**end for**

### Model Implementation

The model was implemented in NetLogo 5.3.1, as well as Python 3.5.2 for external validation. Model runs across multiple parameter settings were conducted using the BehaviourSpace delivered with NetLogo 5.3.1.

Code implementing a single agent is available here:

<https://www.comses.net/codebases/5706/releases/1.1.0/>

On a given run, evidence is generated using the **random-float** function provided by NetLogo to generate binomially distributed 0s and 1s.

The numerical integration necessary to approximate the expected value of the trust function has been taken from the example of Laputa to generate compatible results. The algorithm is a variant of the well-known trapezoid interpolation rule:

$$\int_{0}^{1} \tau_{t}(x)\cong\sum_{i=0}^{n-1} \min\left( \tau_{t}\left( x_{i} \right),\tau_{t\left( x_{i}+1 \right)} \right)*\delta_{x}+0.5*abs\left( \tau_{t}\left( x_{i} \right)-\tau_{t}\left( x_{i}+1 \right) \right)*\delta_{x}$$

where the interpolation distance is for the above simulations chosen to be 1/100, which provides sufficient precision for the parameter configurations simulated.

## Supp. Mat. B: Simulation Details

1. ***Model Parameters***

Here we summarise some further details of the parameters used for the charts shown in the paper.

For the trust-distribution, we used Beta distribution, *beta*(Alpha, Beta), with Alpha = 2 and Beta = 1, resulting in a slightly positive trust *E*(τ) = .66. For the fixed-trust agents we used corresponding fixed values with τ = .66. We chose these values to match the extensive explorations of the Olsson model as an individual agent published in Hahn, Merdes & von Sydow (2018*).* Concerning the hypothesis or claim at issue, we started with belief priors, *P_sub_*(*h*) = .5 and used a hypothesis probability (the probability of an objective occurrence of *h* vs. non-*h*) of *P_obj_*(*h*)=.6. We used 50 sequential time steps for each model run (likewise match the majority of analyses of individual agent behavior reported in Hahn, Merdes & von Sydow, 2018). We recorded the resulting beliefs, squared difference to the true hypothesis value, and various polarisation measures.

For our basic network-type we focused on small world networks (so-called Watts–Strogatz graphs), with *k* = 2 and a rewiring probability of *r* = .2. We chose the number of neighbours as a common value that is widely used with smaller networks and that would be feasible for all values of network size from our smallest network (N=4) to our largest (N=100. In the plots of Fig. 6, however, we additionally used rewiring probabilities of .5 and .8 to vary model structure.

The network size *N* in Fig. 1 to 3 was investigated using *N* = 30, *N* = 60, or *N* = 90. In Fig. 4 to 6 we varied the network size in more detail, starting with *N* = 4, and proceeding from 5 to 100 in steps of 5 (resulting in 21 steps). We chose these values to allow smooth interpolation and extrapolation beyond the computationally feasible boundary of ca. 100 agents. Beyond that, a different implementation and simulation set up for the computationally intensive Olsson model would have to be sought.

The reliability of the evidence (here with the constraint, *p* = *p*(*e*|*h*) = *p*(¬*e*|¬*h*), see Supp. Mat. D.), in Fig. 1 to 5 was varied in three steps (*p* = .55, .66, .75) and in the charts in Fig. 6 running from *p* = .5 to *p* = 1 with steps of .025 (also resulting in 21 steps).

Fig. 1 to 3 show the modelling results for a global activity (‘p-activity’, i.e., the probability of each agent receiving information from the world on given time step) of .1, a threshold of assertation (‘thoa’) that exceeded subjective probability values (i.e., belief or credence values) of .8, and a .25 probability of communication (‘p-communication’) if an agent’s belief has passed this threshold. In Fig. und 5 we investigated different likelihoods either with a high global activity (.50) together with low communication (.20), or with a low global activity (.20) together with a high probability of communication (.50).

Figures are based on a threshold of assertation of .8, but we varied the thresholds of assertation (thoa = .55, .80, .95) in the charts of the Supp. Mat. D.

1. ***Sample Runs***

In order to give readers a better feel for the behavior of the model(s), we show 10 randomly selected runs for the Trust Update agents and the Fixed Trust agents, respectively. Plotted are the belief distributions at time steps 10, 20, 30, 40, and 50. Dark bars indicate the Trust Update agents (blue) and Fixed Trust agents (green), while the light, pink bars show the beliefs of the matched shadow agents. To create these plots, we simply ran the model 10 times for each type of agent and plotted the results. Parameters for these runs were: small world network, *k=* 2*, r =* .2, N=50; *global activity* = .1, *p-comm* = .25; *p_obj(H)*=1, *p_subj(H) = .5; reliability of evidence p =.55, trust beta*(2, 1), τ = .66; *thoa* = .8

**Fig SB1**

**Fig. SB1** Runs 1 to 5 of a 10 run simulation to demonstrate model behavior. Blue bars = update agents, pink bars = shadow agents. For parameters see text Supp Matt B 2. above.

*** Fig SB2**

**Fig. SB2** Runs 6 to 10 of a 10 run simulation to demonstrate model behavior. Blue bars = update agents, pink bars = shadow agents. For parameters see text Supp Matt B 2. above.

***Fig SB3 ***

**Fig. SB3** Runs 1 to 5 of a 10 run simulation to demonstrate model behavior. Green bars = fixed trust agents, pink bars = shadow agents. For parameters see text Supp Matt B 2. above.

***Fig SB4***

**Fig. SB4** Runs 6 to 10 of a 10 run simulation to demonstrate model behavior. Green bars = fixed trust agents, pink bars = shadow agents. For parameters see text Supp Matt B 2. above.

1. **Observations on Sample Model Runs.**

The parameters of the preceding runs were chosen to emphasise the impact of communication (by choosing a higher value for the communication probability) while also emphasising the divergence between models (in particular with respect to polarisation) by setting the quality of the evidence from the world to be poor (*p* = .55, the lowest level shown in Figs. 1 to 5 main text). The preceding sample runs illustrate several features of the models:

1. The expectation-based update strategy creates a greater dispersion in degrees of belief: to see this, contrast shadow agents at Steps 30, 40, 50 for the trust update agents (Fig SB1. and SB2) with Steps 30, 40, 50 for the fixed trust agents (Fig. SB3 and SB4), in keeping with the findings of Hahn, Merdes & von Sydow, 2018.
2. Belief divergence is faster among update agents than it is for fixed trust agents: to see this, contrast Step 20 between trust update and fixed trust agents.
3. The erosion of the middle seems more pronounced in the update agents, even within this small sample: see Step 40.
4. Under these parameter settings, there is a high chance of polarisation at Step 50 for both update and fixed trust agents. The relative size of the extremal groups varies from a polarised minority to roughly equal splits.
5. A higher rate of polarisation among the update agents is apparent even in this small sample: under the ‘minimal’ polarization measure used in the main text (at least one agent at each extreme end of the belief spectrum), runs 1,2,3 (Fig. SB1) and 6,7,8,9,10 (Fig. SB2) for the update agents would be classified as resulting in a ‘polarised’ society, versus runs 4,5 (Fig. SB3) and 7,8,10 (Fig. SB4) for the fixed trust agents.
6. Finally, while there is a strong tendency for the majority of networked agents to converge toward the true state of the world (Hypothesis = 1 in all graphs shown here), there exist runs where ‘herding’ and ‘bandwagon-ing’ lead the majority to the wrong conclusion (see also Hahn, Hansen, Olsson, 2020).

## Supp. Mat. C: Supplementary Polarisation Material

### 1. Note: On the Difficulties of Measuring Polarisation

As we detail in the main text (Section “The Findings in Context”) research interest in polarisation spans a wide range of disciplines and contexts. It is consequently not surprising that ‘polarisation’ has a family resemblance structure with an overlapping set of more specific meanings (see e.g., Bramson et al., 2016 who distinguish six different senses of the word). In light of that, it is also no surprise that there is no single, agreed upon measure of polarisation in the literature. The difficulties with measuring polarisation, however, go beyond conceptual heterogeneity of target notions. As Esteban and Ray (1994) , detail, an appropriate measure of polarisation presents a considerable formalisation challenge because -even for a conceptually clear notion- there are multiple individual factors for which the desired trade off in an overall measure is unclear: arguably, a measure of polarisation should capture the degree to which communities are separate, and the degree to which these separate communities are themselves homogenous, and it should be sensitive to the number of individuals in each group (such that two equal groups score highest). Combining these different intuitions into a single measure in non-trivial and admits of multiple solutions. In particular, there is no unique way to weight these different factors as they trade off: for example, should two groups that are closer but more equal in size be viewed as more, less, or equally polarised than a group that has greater between group distance, but a slightly more unequal balance in the size of the respective groups? Furthermore, there is an additional requirement that plays a role in the context of the specific investigation we are conducting (which examines polarisation as a function of network size), namely that the measure be normalised or at least well-behaved with respect to *population size*.

We illustrate these complexities with respect to the polarisation measure employed by Olsson (2013) in the initial examination of polarisation with rational agents using the Olsson (2011) model: the standard deviation. As indicated in the main text, we calculated this measure, among other things, to enable comparison with that work. Figure SC1 below shows the results of using the standard deviation as a measure of polarisation for the data in Figures 4 and 5, main text.

What emerges clearly from that figure is two of the main findings of our paper namely that 1) expectation-based updating produces more polarisation than a fixed trust strategy, and that 2) polarisation increases as a function of network size. What is not readily apparent, however, is our other main finding: that communication across social networks increases polarisation. The standard deviation of the populations of non-communicating shadow agents (right hand columns) are high throughout, which would suggest a high degree of polarisation that is totally at odds with the sample simulation runs in Figures SB1 to SB4 above, where none of the Step 50 shadow distributions show meaningful segregation into two groups. This is due to the mathematical properties of the standard deviation and the way it interacts with the belief distributions generated by the different types of agents.

We illustrate these below in Fig. SC2. This figure visualises the standard deviation for populations of size N=10, 20, 40, 60, 80, and 100, respectively. As can be seen, even what seem intuitively like comparable cases (e.g., a population consisting only of extreme beliefs (0 or 1) with both groups equal in size (see top left panel) are not equal in polarisation on this measure. Instead, polarisation drops as a function of population size (in contrast to what is observed in our data Fig. SC1).

**Fig. SC 1** Polarisation as Standard Deviation

*****Fig SC1 *****

Fig. SC1: Mean standard deviation of the beliefs in the simulation runs; shown for different net size and varied activity and communication levels with the same model parameters as Figure 4 and 5 (see main text and Supp. Mat. B for further parameter details). The upper dotted line with greater polarisation corresponds to *p* = .55, the continuous line in the middle represents *p* = .66, the lowest dotted to *p* = .75. The area between the two dotted lines is shaded in the corresponding color.

This makes the standard deviation less than ideal for comparing across populations of different sizes, as we do in our project here. The standard deviation does, however, behave in intuitive ways regarding the relative size of two maximally separated clusters in that it is highest for a population with extremal beliefs that is split exactly in half (see top left panel Fig. SC2), and drops as one group becomes larger and larger by comparison (top right hand panel, Fig. SC2, bottom left panel, Fig SC2).

**Fig. SC 2** Polarisation as Standard Deviation Across Different Distribution Types

*****Fig SC 2*****

Fig. SC2: The figure shows the s.d. for different hypothetical belief distributions: top left, extremal populations of size N comprised solely of 0s and 1s with a of 50/50% split in terms of frequency; top right, the same with 30/70, 20/80, and 10/90 splits of 0s to 1s; bottom left a single contrarian (0) in a population of 1s; and bottom right, a uniform distribution that partitions the interval 0 to 1 in N (= population size) unique, equally spaced distances.

However, a population of 1s and 0s with a 90/10 split (top right panel, Fig. SC2) has almost the same standard deviation as a uniform distribution (bottom right panel, Fig. SC2). More generally, the standard deviation becomes opaque when it comes to understanding the trade-offs between the quantities that go into shaping intuitions about degree of polarisation: the between cluster distance, the within cluster distance, the relative cluster sizes, and the extent to which there may remain a scattering of agents between clusters that might ‘bridge’ them.

Furthermore, the relative importance one might attach to each of these factors may vary with context of interest.

For example, what gets labelled a ‘conspiracy theory’ in real life is inherently a view held only by a minority. Yet the extreme epistemic divergence presented by conspiracy theorists raises issues of both theoretical and practical concern (which is why we include discussion of them in section “The Findings in Context” above). But adopting a cut-off for when a minority seems ‘large enough’ for the group to count as polarised seems arbitrary, as does assigning a relative weight to comparative group size relative to intra-cluster distance.

In light of these conceptual difficulties for any measure of polarisation, and in the absence of a single, agreed upon measure in the research literature, the question that must be asked in selecting a measure is simply whether the chosen measures are fit for purpose in underscoring the theoretical conclusions at issue (in the specific context). Moreover, it makes sense to employ multiple measures to capture the distinct components of polarisation as recommended by Bramson et al. (2016, pg. 105).

To this end, we chose to combine three simple measures which, though individually limited, together confirm our findings to be robust:

**Measure 1, our ‘Min Polarisation’ measure shows that there are extremists (without seeking to measure the relative group size, or the presence of an intermediate ‘gap’). Measure 2, our ‘Max gap’ measure, rules out uniform distribution by showing that there are gaps. Finally, Measure 3, the standard deviation, shows that the members of the extremal group are not just isolates or ‘lone contrarians’ as can be seen by comparing the actual data in the left hand column of Fig. SC1 with the ‘contrarian’ distribution of SC2 bottom left panel.**

Together, these three measures thus provide a robust confirmation of what is suggested by the sample runs SB1-SB4, and what we take to be the key insights of this paper: namely that communication across a network promotes polarisation, that expectation-based updating increases the polarisation risk, and that that risk increases as a function of network size.

## Supp. Mat. D: Robustness & Supplementary Simulations

### 1. Symmetry Assumption

Motivation for symmetry assumption, and limitations of the symmetry assumption: Symmetry means that the evidential impact of a positive and a negative piece of evidence are equal and, in overall effect, cancel one another out. Relaxing symmetry simply relaxes this constraint. In the absence of specific information about sensitivity and specificity, as is the case in our problem by definition, assuming symmetry seems appropriate, reflecting a type of ‘indifference’.

It also enables straightforward Bayesian updating, which would not be possible if sensitivity, *P*(*e*|*h*), and specificity, *P*(¬*e*|¬*h*), were free to vary, as there would then be two parameters to adjust in response to each data point, leaving the expectation-based revision problem under-determined. One of the two values would thus have to be fixed. At the same time, this would change only the relative impact of positive versus negative evidence, but crucially not the range of LHR’s that are possible for an agent to entertain. As set out in the main text and by Eq. 2, this means also that all posterior degrees of belief are possible as the LHR is the only quantity that is relevant for this. Relaxing symmetry would consequently not bring more generality in this regard. It would also not alter any of the structural challenges of the trust update problem as set out in Figure 4 main text.

### 2. Threshold of Assertion

Since we only used one threshold of assertion value modelling in the main paper (thoa = .8), we conducted additional robustness checks by varying this threshold (.55, .80, .95), here using a global activity (‘p-activity’) level of .1 and a probability of communication of .25. As in the other simulations we used a belief prior, *P_sub_*(*h*) = .5, and a trust prior Beta distribution *beta*(Alpha, Beta), with Alpha = 2, Beta = 1, and a resulting E(τ) = .66. For a fixed trust we used τ = .66. For the other parameters see the values given in Supp. Mat. B.

#### Accuracy: Mean Square Difference

Supp. Fig. SD 1

***Fig SD 1***

Fig. SD1: (In)accuracy as mean squared difference from the true value of the hypothesis (0 or 1), shown for different net sizes and levels of threshold of assertion (see main text and Supp. Mat. B for further parameter details). The upper dotted line with greater error corresponds to *p* = .55, the continuous line in the middle represents *p* = .66, the lowest dotted to *p* = .75. The area between the two dotted lines is shaded in the corresponding color.

#### Minimal Polarisation

Supp. Fig. SD 2

***Fig SD 2 ***

Fig. SD2: Mean Minimal Polarisation values shown for different net sizes and levels of “threshold of assertion” (see main text and Supp. Mat. B for further parameter details). The upper dotted line with greater polarisation corresponds to *p* = .55, the continuous line in the middle represents *p* = .66, the lowest dotted to *p* = .75. The area between the two dotted lines is shaded in the corresponding color.

#### Max-Gap Polarisation

Supp. Fig. SD 3

***Fig SD 3***

Fig. SD3: Mean Max-Gap-Polarisation values shown for different net sizes and levels of threshold of assertion (see main text and Supp. Mat. B for parameter details). The upper dotted line with greater polarisation corresponds to *p* = .55, the continuous line in the middle represents *p* = .66, the lowest dotted to *p* = .75. The area between the two dotted lines is shaded in the corresponding color.

References

Bramson, A., Grim, P., Singer, D. J., Fisher, S., Berger, W., Sack, G., & Flocken, C. (2016). Disambiguation of social polarization concepts and measures. *The Journal of Mathematical Sociology*, *40*(2), 80-111.

Esteban, J. M., & Ray, D. (1994). On the measurement of polarisation. *Econometrica: Journal of the Econometric Society*, 819-851.

Flache, A., & Mäs, M. (2015). Multi-Agenten-Modelle. In *Handbuch Modellbildung und Simulation in den Sozialwissenschaften* (pp. 491-514). Springer VS, Wiesbaden.

Hahn, U., Hansen, J.U. & Olsson, E.J. (2020) Truth tracking performance of social networks: how connectivity and clustering can make groups less competent. *Synthese, 197* (4), 1511-1541.

Hahn, U., Merdes, C. & von Sydow, M. (2018). How Good is Your Evidence and How Would You Know? *Topics in Cognitive Science, 10,* 660-678.

Olsson, E. J. (2013). A Bayesian simulation model of group deliberation and polarisation. In *Bayesian Argumentation* (pp. 113-133). Springer Netherlands.
